# Diverse mitochondrial abnormalities in a new cellular model of *TAFFAZZIN* deficiency are remediated by cardiolipin-interacting small molecules

**DOI:** 10.1016/j.jbc.2021.101005

**Published:** 2021-07-24

**Authors:** Arianna F. Anzmann, Olivia L. Sniezek, Alexandra Pado, Veronica Busa, Frédéric M. Vaz, Simion D. Kreimer, Lauren R. DeVine, Robert N. Cole, Anne Le, Brian J. Kirsch, Steven M. Claypool, Hilary J. Vernon

**Affiliations:** 1 Department of Genetic Medicine, Johns Hopkins University School of Medicine, Baltimore, Maryland, USA; 2 Department of Clinical Chemistry and Pediatrics, Academic Medical Center, Amsterdam, The Netherlands; 3 Mass Spectrometry and Proteomics Facility, Department of Biological Chemistry, Johns Hopkins University School of Medicine, Baltimore, Maryland, USA; 4 Department of Pathology and Oncology, Johns Hopkins University School of Medicine, Baltimore, Maryland, USA; 5 Department of Physiology, Johns Hopkins University School of Medicine, Baltimore, Maryland, USA

**Keywords:** cardiolipin, mitochondrial metabolism, Barth syndrome, BEL, bromoenol lactone, BN-PAGE, blue native-PAGE, BTHS, Barth syndrome, CCCP, carbonyl cyanide *m*-chlorophenyl hydrazine, cDNA, complementary DNA, CI, complex I, CII, complex II, CIV, complex IV, CL, cardiolipin, FC, fold change, GO, Gene Ontology, HEK293, human embryonic kidney 293 cell, IMM, inner mitochondrial membrane, KEGG, Kyoto Encyclopedia of Genes and Genomes, MLCL, monolysocardiolipin, MQC, mitochondrial quality control, OXPHOS, oxidative phosphorylation, PARL, presenilin-associated rhomboid-like protein, PBST, PBS with 0.2% (v/v) Tween-20, PGAM5, phosphoglycerate mutase 5, sgRNA, single-guide RNA, TAZ, TAFFAZIN

## Abstract

Barth syndrome (BTHS) is an X-linked disorder of mitochondrial phospholipid metabolism caused by pathogenic variants in *TAFFAZIN*, which results in abnormal cardiolipin (CL) content in the inner mitochondrial membrane. To identify unappreciated pathways of mitochondrial dysfunction in BTHS, we utilized an unbiased proteomics strategy and identified that complex I (CI) of the mitochondrial respiratory chain and the mitochondrial quality control protease presenilin-associated rhomboid-like protein (PARL) are altered in a new HEK293–based tafazzin-deficiency model. Follow-up studies confirmed decreased steady state levels of specific CI subunits and an assembly factor in the absence of tafazzin; this decrease is in part based on decreased transcription and results in reduced CI assembly and function. PARL, a rhomboid protease associated with the inner mitochondrial membrane with a role in the mitochondrial response to stress, such as mitochondrial membrane depolarization, is increased in tafazzin-deficient cells. The increased abundance of PARL correlates with augmented processing of a downstream target, phosphoglycerate mutase 5, at baseline and in response to mitochondrial depolarization. To clarify the relationship between abnormal CL content, CI levels, and increased PARL expression that occurs when tafazzin is missing, we used blue-native PAGE and gene expression analysis to determine that these defects are remediated by SS-31 and bromoenol lactone, pharmacologic agents that bind CL or inhibit CL deacylation, respectively. These findings have the potential to enhance our understanding of the cardiac pathology of BTHS, where defective mitochondrial quality control and CI dysfunction have well-recognized roles in the pathology of diverse forms of cardiac dysfunction.

Barth syndrome (BTHS; Mendelian Inheritance in Man accession number: 302060) is a rare X-linked inborn error of mitochondrial phospholipid metabolism caused by pathogenic variants in the gene *TAFAZZIN* (1, 2, 3). *TAFAZZIN* encodes a transacylase essential for the remodeling and maturation of the mitochondrial phospholipid cardiolipin (CL) (1, 4). CL, primarily localized to inner mitochondrial membrane (IMM), has many key functions, including roles in maintaining mitochondrial cristae structure, organization of respiratory complexes, protein import, fusion, and cellular signaling (5, 6, 7). Tafazzin deficiency results in abnormal CL content, including an accumulation of the remodeling intermediate monolysocardiolipin (MLCL), decreased remodeled CL, and a shift toward saturated CL species (1, 5). An elevated MLCL:CL ratio is the pathognomonic metabolic defect in BTHS and is found in 100% of affected individuals (8).

BTHS is a multisystem disorder characterized by prenatal onset of left ventricular noncompaction, early onset cardiomyopathy, skeletal myopathy, growth abnormalities, and neutropenia among other features and is the only known Mendelian disorder of CL metabolism (9, 10, 11). Despite knowledge of the primary metabolic defect in BTHS, there is limited knowledge of downstream mechanisms of cellular pathogenesis, and consequently, there is a dearth of targets for therapeutic intervention and clinical monitoring (12). In addition to BTHS, CL abnormalities have been described in common conditions such as idiopathic cardiomyopathy, fatty liver disease, and diabetes (13, 14, 15, 16). Consequently, studies in BTHS have the potential to illuminate pathophysiology in a range of common diseases.

We performed shotgun proteomics on a new model of tafazzin deficiency in human embryonic kidney 293 (HEK293) cells developed and validated in our laboratory to identify and clarify pathways of mitochondrial dysregulation. We found that steady-state levels of specific subunits of complex I (CI) are decreased in tafazzin deficiency and are associated with abnormal CI function and assembly. We further identified elevated abundance of presenilin-associated rhomboid-like protein (PARL), a rhomboid protease associated with the mitochondrial membrane response to stress, and observed increased cleavage of a downstream protease target, phosphoglycerate mutase 5 (PGAM5), in response to mitochondrial depolarization. Importantly, we showed that small-molecule modifiers of CL metabolism remediate both CI defects and PARL overexpression in tafazzin deficiency. These findings are highly relevant to the cardiac pathology of BTHS, as defective mitochondrial quality control (MQC) and mitochondrial CI defects are increasingly recognized as playing important roles in diverse forms of cardiac dysfunction (17, 18, 19) and suggest specific therapeutic approaches.

## Results

### Generation of a HEK293 *TAFAZZIN*-deficient model, *TAZ*^Δ45^

**Figure 1:**
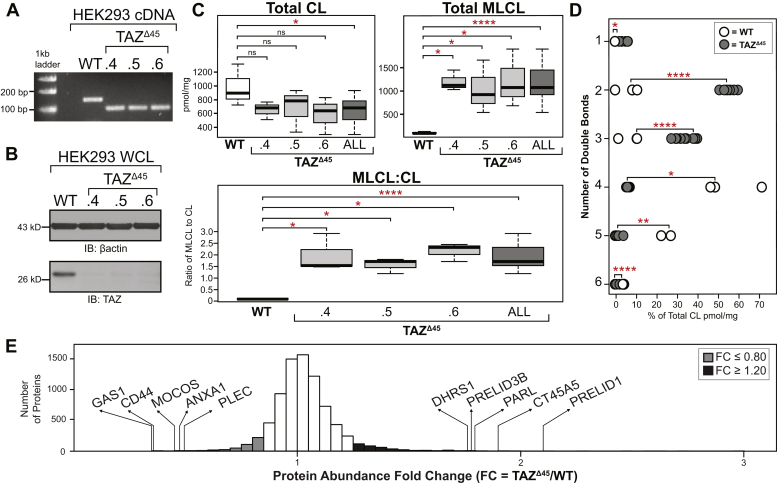
***TAZ***^**Δ45**^**HEK293 genetic characterization, CL profiling, and proteomics analysis.***A,* RT-PCR of mRNA extracted from the HEK293 *TAZ*^Δ45^ clones using primers specific to the region of *TAZ* being edited. *B,* whole cell lysates (45 μg) of the indicated cell lines immunoblotted for tafazzin and loading control β-actin. *C,* the abundance of CL and MLCL in WT (n = 3) and *TAZ*^Δ45^ clones (n = 3, per clone) was determined by shotgun lipidomics *via* MS. *D,* the distribution of double bonds across CL species was determined as a percentage of total CL. *E,* 7713 proteins were identified and quantified with tandem mass tag 10-plex MS of whole cell lysate (200 μg) of *TAZ*^Δ45^ (n = 3) and WT cells (n = 3). Protein abundance fold change (FC) is calculated by dividing the average abundance per protein identified in *TAZ*^Δ45^ cells by WT average abundance per protein. The genes that encode the most significantly reduced proteins in *TAZ*^Δ45^ cells are *GAS1* (FC = 0.35; *p* = 2.5 × 10^−4^), *CD44* (FC = 0.367; *p* = 6.6 × 10^−4^), *MOCOS* (FC = 0.45; *p* = 0.011), *ANAX1* (FC = 0.478; *p* = 0.01), and *PLEC* (FC = 0.491; *p* = 2.7 × 10^−4^). The genes that encode the most significantly increased proteins in *TAZ*^Δ45^ cells are *PRELID1* (FC = 2.125; *p* = 8.4 × 10^−4^), *CT45A5* (FC = 1.909; *p* = 0.023), *PARL* (FC = 1.815; *p* = 0.016), *PRELID3B* (FC = 1.795; *p* = 3.9 × 10^−4^), and *DHRS1* (FC = 1.774; *p* = 0.016). Proteins with an FC ≤ 0.80 are highlighted in *gray* (n = 215), and proteins with an FC ≥ 1.20 are highlighted in *black* (n = 621). Significant differences are indicated; ∗≤0.05, ∗∗≤0.005, ∗∗∗≤0.0005, and ∗∗∗∗≤0.00005. CL, cardiolipin; HEK293, human embryonic kidney 293 cells; MLCL, monolysocardiolipin; *TAZ*, TAFFAZIN.

We used CRISPR–Cas9 genome editing in HEK293 cells to create a novel *TAFAZZIN*-deficient cellular model. Using two single-guide RNAs (sgRNAs) targeting exon 2 of *TAFAZZIN*, we isolated three clones with a 45 bp deletion at the 3′-end of exon 2; *TAZ*^Δ45.4^, *TAZ*^Δ45.5^, and *TAZ*^Δ45.6^ (Fig. S1 and Table S1). The deletion encompasses a predicted acyltransferase domain and covers an area of *TAFAZZIN* where multiple pathological variants in patients with BTHS have been described (3). The 45 bp in-frame deletion is not predicted to result in nonsense mediated decay, and mRNA expression was detected at an intensity comparable to WT but at a smaller size (Fig. 1*A*). However, the deletion resulted in undetectable expression of tafazzin protein in all three clones (Fig. 1*B*). In the absence of tafazzin, there was no significant difference in the abundance of cytosolic (β-actin) and mitochondrial proteins (glucose-regulated protein 75, voltage-dependent anion channel 1, and translocase of the outer membrane 20) (Fig. S2).

Shotgun lipidomics *via* MS revealed a significant decrease in CL, a significant increase in MLCL, and a significantly increased MLCL:CL ratio (*p* = 0.03, *p* = 4.9 × 10^−5^, *p* = 4.6 × 10^−6^, respectively) (Fig. 1*C*). Tafazzin-based remodeling is characterized by the incorporation of unsaturated acyl chains compared with nascent CL. Of the 31 CL species assessed, the *TAZ*^Δ45^ clones had a significant increase in CL containing one to three double bonds (*p* = 0.007, *p* = 1.5 × 10^−9^, *p* = 7.4 × 10^−6^, respectively) and a significant decrease in CL containing four to six double bonds (*p* = 0.03, *p* = 0.003, *p* = 4.2 × 10^−9^, respectively), highlighting the loss of tafazzin-based remodeling (Fig. 1*D*). Collectively, the *TAZ*^Δ45^ clones recapitulate the pathognomonic metabolic defect of BTHS and other well-published cellular models of BTHS (2) and validate the *TAZ*^Δ45^ clones as cellular models of BTHS.

### Differentially abundant proteins in *TAZ*^Δ45^ cells reveal downstream cellular dysfunction because of *TAFAZZIN* deficiency

Shotgun proteomics analysis identified a total of 7713 proteins in WT and *TAZ*^Δ45^ cells. To focus our downstream workflow on proteins with differential abundance between the WT and *TAZ*^Δ45^ cells, we selected proteins with a protein abundance fold change (FC; *TAZ*^Δ45^/WT) less than or equal to 0.80 (FC ≤ 0.80) and proteins with an FC greater than or equal to 1.2 (FC ≥ 1.20) (Fig. 1*E*). Based on these criteria, there were a total of 836 differentially abundant proteins, 215 with an FC ≤ 0.80 and 621 with an FC ≥ 1.20 (Fig. 1*E*). Functional annotation of the differentially abundant proteins, with Kyoto Encyclopedia of Genes and Genomes (KEGG) pathway and Gene Ontology (GO) term enrichment analysis, identified multiple pathways of interest in *TAZ*^Δ45^ cells (Table S2, *A* and *B*) (18, 19).

### Functional annotation and analysis of proteins with decreased expression: proteins of respiratory CI

We identified 86 significantly enriched (*p* ≤ 0.05) KEGG pathways and GO terms for proteins with an FC ≤ 0.80, such as oxidative phosphorylation (OXPHOS) (*p* = 2.7 × 10^−6^), mitochondrial respiratory chain CI assembly (*p* = 7.9 × 10^−5^), mitochondrial chain CI (*p* = 2.1 × 10^−4^), response to oxidative stress (*p* = 1.6 × 10^−3^), NADH dehydrogenase (ubiquinone) activity (*p* = 2.1 × 10^−3^), and metabolic pathways (*p* = 0.019) (Table S2*A*). Defects in OXPHOS function have been previously described in BTHS, and consistent with these previous studies, we found that of the 86 terms significantly enriched for proteins with an FC ≤ 0.80 in *TAZ*^Δ45^ cells, 18 reference the mitochondrion and/or OXPHOS (Table S2*A*). Specifically, 11 proteins with an FC ≤ 0.80 are encoded by genes associated with the OXPHOS KEGG pathway (Fig. S3). Of these 11 proteins, five are subunits of CI and the remaining six proteins are subunits of complexes III, IV, and V (Fig. S3). When we further examined the 18 terms that reference the mitochondrion and/or OXPHOS, we found that four specifically reference CI of OXPHOS (Table S2*A*).

**Figure 2:**
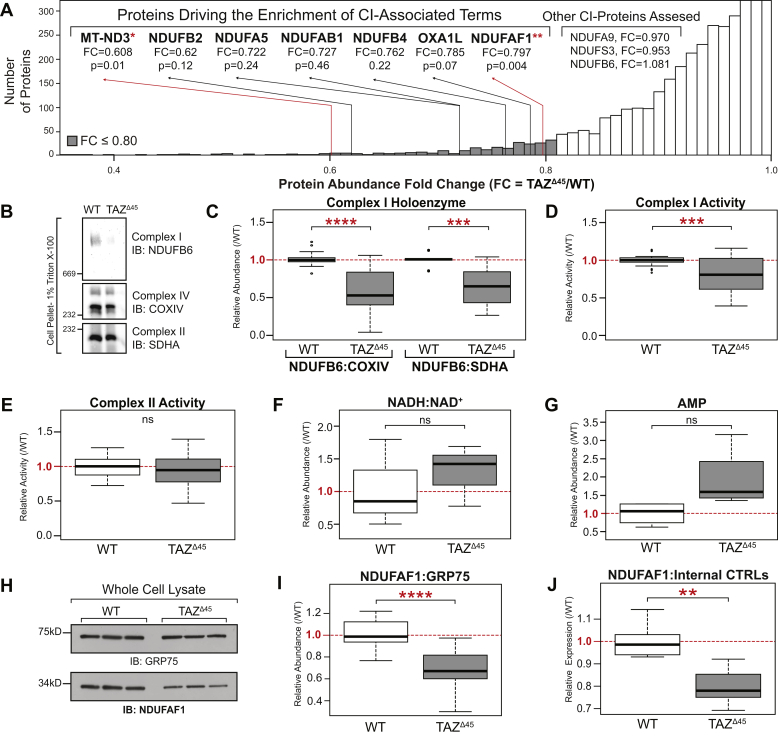
**Reduced complex I (CI) holoenzyme abundance and activity in HEK293 *TAZ***^**Δ45**^**cells.***A,* the enrichment of the CI-associated terms is driven by seven genes; *MT-ND3*, *NDUFAF1*, *NDUFA5*, *NDUFAB1*, *NDUFB2*, *NDUFB4*, and *OXA1L*. *B,* BN-PAGE of HEK293 WT and *TAZ*^Δ45^ (100–250 K cells) solubilized in 1% Triton X-100 immunoblotted for the indicated proteins. *C,* band intensities were quantified, and CI abundance was represented as the ratio of CI band intensity to CIV (COXIV) or CII (SDHA). Abundance was plotted relative to WT/CTRL abundance; CI:CIV (WT, n = 13; *TAZ*^Δ45^, n = 16), CI:CII (WT, n = 10; *TAZ*^Δ45^, n = 13). *D,* CI activity measured in HEK293 WT and *TAZ*^Δ45^ mitochondria (200 μg total protein) on a microplate reader (450 nm) by following the oxidation of NADH to oxidized NAD^+^. Activity plotted relative to WT/CTRL abundance (WT, n = 25; *TAZ*^Δ45^, n = 26). *E,* CII activity measured in HEK293 WT and *TAZ*^Δ45^ mitochondria (200 μg total protein) on a microplate reader by following the production of ubiquinol by CII coupled to the reduction of the dye diclorophenolindophenol (DCPIP; 600 nm). Activity plotted relative to WT/CTRL abundance (WT, n = 12; *TAZ*^Δ45^, n = 12). Targeted metabolomics was used to measure (*F*) NADH, NAD^+^, and (*G*) cellular AMP *via* MS in HEK293 WT (n = 3) and HEK293 *TAZ*^Δ45^ (n = 3) cells. *H,* whole cell lysates (45 μg) of the indicated lines were immunoblotted for the indicated proteins. *I,* band intensities, relative to the loading control GRP75, were quantified and plotted relative to WT/CTRL abundance. *J,* relative mRNA expression of *NUDFAF1*, determined by qRT-PCR and ΔΔC_T_ quantification. Data plotted relative to WT/CTRL expression (WT, n = 6; *TAZ*^Δ45^, n = 6; WT, n = 27; *TAZ*^Δ45^, n = 26). Significant differences are indicated; ∗≤0.05, ∗∗≤0.005, ∗∗∗≤0.0005, and ∗∗∗∗≤0.00005. GRP75, glucose-regulated protein 75; HEK293, human embryonic kidney 293 cells; TAZ, TAFFAZIN.

The enrichment of the CI-associated terms in our functional annotation analysis was driven by seven proteins encoded by the genes: *MT-ND3*, *NDUFAF1*, *NDUFA5*, *NDUFAB1*, *NDUFB2*, *NDUFB4*, and *OXA1L* (Fig. 2*A*). Five are subunits of CI (*MT-ND3*, *NDUFA5*, *NDUFAB1*, *NDUFB2*, and *NDUFB4*), one is a CI assembly factor (*NDUFAF1*), and one assists with inserting proteins into the IMM and has been implicated in CI biogenesis (*OXA1L*) (20, 21).

Examination of the proteomics data for expression and abundance of each individual protein involved with CI function showed that 46 of the 56 identified/quantified CI proteins had reduced abundance in *TAZ*^Δ45^ cells compared with WT (FC range = 0.608–0.998) (Table S3), highlighting that while the CI association identified by functional annotation was driven by seven proteins, the total proteomics data showed an overall decrease in expression of most CI-associated proteins.

### Functional annotation and analysis of proteins with increased expression: proteins of mitochondrial stress response

We identified 127 significantly enriched (*p* ≤ 0.05) KEGG pathways and GO terms for proteins with an FC ≥ 1.20 and found that 20 terms reference the mitochondrion or mitochondrial dynamics, including metabolic pathways (*p* = 3.1 × 10^−3^), positive regulation of the apoptotic process (*p* = 4.2 × 10^−3^), and IMM (*p* = 5.2 × 10^−3^) (Table S2*B*). The functional annotation analysis revealed other genes of interest because of their potential roles in apoptosis, lipid trafficking, and/or MQC, such as PRELID1, PRELID3B, CASP2, CASP7, CASP8, and CASP9 (22, 23, 24). PARL was among the top five proteins with significantly increased abundance in the proteomics data providing further evidence of dysregulation in associated pathways (Fig. 1*E*). PARL has been implicated in regulating the mitochondrial responses to stress, including membrane depolarization and increased reactive oxygen species (25, 26, 27), and dysregulation of PARL substrates has been implicated in cardiomyopathy and cardiac development (28, 29, 30). Together with the proteomics findings, the functional annotation analysis suggests an increased abundance of proteins involved in the mitochondrial stress response, a pathway not previously described in BTHS.

### Reduced CI holoenzyme and activity in HEK293 *TAZ*^Δ45^ cells

Because of the decrease in CI-associated proteins uncovered *via* proteomics analysis, we investigated the overall function and assembly of CI. To determine the total abundance of individual respiratory complexes including the CI holoenzyme, HEK293 cells were solubilized with Triton X-100 and resolved by blue native-PAGE (BN-PAGE). CI was the most significantly reduced complex, and the ratio of CI to complex IV (CIV) (CI:CIV) or CI to complex II (CII) (CI:CII) was significantly reduced in *TAZ*^Δ45^ *versus* WT (*p* = 2.32 × 10^−5^, *p* = 1.62 × 10^−4^, respectively) (Fig. 2, *B* and *C*). To verify that the observed differences in CI abundance were not because of an effect of altered CL on Triton X-100 solubilization, we compared starting material and the cellular pellet following solubilization *via* immunoblotting and found no significant difference in CI solubilization in *TAZ*^Δ45^ and WT cells. In order to determine whether the effects of tafazzin deficiency were specific to CI, we immunoblotted for a subunit of respiratory complex III (for ubiquinol–cyctochrome C reductase core protein 2) and found no significant difference in abundance between WT and *TAZ*^Δ45^ cells (Fig. S5).

We next demonstrated a significant reduction in CI enzyme activity in *TAZ*^Δ45^ cells compared with WT (*p* = 9.4 × 10^−5^) using a colorimetric CI enzyme activity assay that detects the oxidation of NADH to NAD^+^ (Fig. 2*D*). There was no significant difference in CII activity between WT and *TAZ*^Δ45^ cells, emphasizing the specificity for CI, as opposed to generalized respiratory chain dysfunction, which occurs when tafazzin is lacking (Fig. 2*E*).

Measurement of intracellular NADH and NAD+ showed a trend toward an increase in the ratio of NADH to NAD^+^ in *TAZ*^Δ45^ compared with WT, though this did not reach statistical significance (Fig. 2*F*). There was also an increase in intracellular AMP in *TAZ*^Δ45^ cells compared with WT (Fig. 2*G*). This disturbance in energy homeostasis is consistent with the decreased CI expression and activity in tafazzin -deficient cells.

### CI subunit and assembly factor expression in *TAZ*^*Δ*45^ cells

The most significantly reduced CI-associated protein identified in the proteomics data was NDUFAF1 (*p* = 0.004). NDUFAF1 is a CI assembly factor, involved in the stepwise construction of CI assembly modules (31). Defects in NDUFAF1 have been shown to cause infantile-onset cardiomyopathy, CI enzymatic defects, and CI assembly defects (19). Thus, decreased expression of this protein represented a strong candidate for playing a major role in the abnormal expression of CI and CI function in tafazzin*-*deficient cells. Immunoblotting of HEK293 whole cell lysate for NDUFAF1 confirmed reduced protein abundance in *TAZ*^Δ45^ cells (FC = 0.69; *p* = 4.89 × 10^−10^) (Fig. 2, *H* and *I*). Decreased mRNA expression of *NDUFAF1* was also seen in *TAZ*^Δ45^ cells (FC = 0.80; *p* = 6.4 × 10^−4^) (Fig. 2*J*). To determine if rescuing decreased NDUFAF1 would restore CI dysfunction in tafazzin-deficient cells, we overexpressed NDUFAF1 in *TAZ*^Δ45^ cells. Overexpression of NDUFAF1 did not normalize the CI functional deficiency in *TAZ*^Δ45^ cells (Fig. S6, *A–C*), prompting further investigation into the other CI subunits and assembly factors shown to be altered *via* proteomics in tafazzin-deficient cells.

We next measured the relative mRNA expression of the five other CI-associated proteins that had a reduced protein expression (FC ≤ 0.80) in the proteomics analysis (NDUFB2, NDUFAB1, NDUFB4, MT-ND3, and NDUFA5) and three additional CI-associated proteins (NDUFA9, NDUFS3, and NDUFB6), which did not have an FC ≤ 0.80 but had been previously shown to have reduced protein abundance in another tafazzin-deficient cellular model (Fig. S4) (17). Of these eight genes, four had significantly reduced mRNA expression in *TAZ*^Δ45^ cells (*NDUFB2*, *p* = 0.04; *NDUFAB1*, *p* = 0.001; *NDUFB4*, *p* = 0.02; and *NDUFB6*, *p* = 0.01) (Fig. S4). *NDUFA5*, *NDUFA9*, and *NDUFS3* had reduced mRNA expression in *TAZ*^Δ45^ cells although they did not reach statistical significance (Fig. S4).

Thus, we identified a CI function- and expression-specific respiratory chain defect in tafazzin-deficient HEK293 cells, driven by both abnormal gene and protein expression of CI subunits and assembly factors. Our results suggest that of the five OXPHOS complexes, CI expression and function is uniquely sensitive to the absence of tafazzin-based CL remodeling. A limited number of studies in patient-derived lymphoblasts have also suggested a special role for CI dysfunction in tafazzin deficiency, highlighting the relevance of these findings for human disease (32, 33, 34).

### Abnormal PARL abundance and cleavage of its downstream target PGAM5

**Figure 3:**
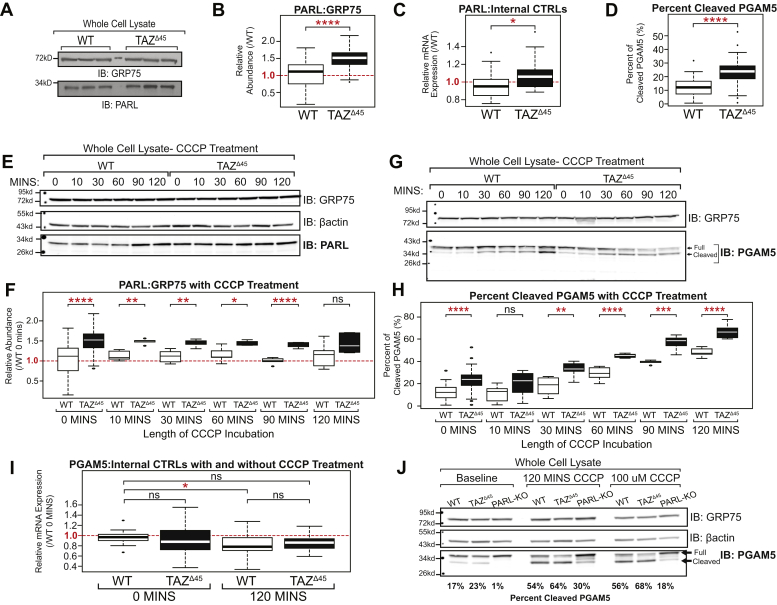
**Increased cleavage of PGAM5 by PARL in *TAZ***^**Δ45**^**cells.***A,* whole cell lysates (45 μg) of the indicated lines were immunoblotted for the indicated proteins. *B,* band intensities, relative to loading control GRP75, were quantified and plotted relative to WT abundance (WT, n = 54; *TAZ*^Δ45^, n = 48). *C,* relative mRNA expression of *PARL* determined by qRT-PCR and ΔΔC_T_ quantification (WT, n = 18; *TAZ*^Δ45^, n = 17). *D,* whole cell lysates (45 μg) of WT and *TAZ*^Δ45^ cells were immunoblotted for PGAM5 and loading control GRP75. Band intensities, relative to the loading control GRP75, for both full-length and cleaved PGAM5 were individually quantified and plotted as the percent of cleaved PGAM5 (cleaved/full + cleaved) (WT, n = 41; *TAZ*^Δ45^, n = 41). *E,* HEK293 WT and *TAZ*^Δ45^ cells were treated with 20 μM CCCP for serial time points from 0 to 120 min. Whole cell lysates (45 μg) of the indicated lines and treatment times were immunoblotted for the indicated proteins. Densitometry quantification can be found in Figure S4. *F,* band intensities, relative to the loading control GRP75, were quantified and plotted relative to WT abundance; WT 0 min n = 54, WT 10 min n = 5, WT 30 min n = 6, WT 60 min n = 6, WT 90 min n = 5, WT 120 min n = 6, *TAZ*^Δ45^ 0 min n = 48, *TAZ*^Δ45^ 10 min n = 5, *TAZ*^Δ45^ 30 min n = 5, *TAZ*^Δ45^ 60 min n = 5, *TAZ*^Δ45^ 90 min n = 5, and *TAZ*^Δ45^ 120 min n = 6. *G,* HEK293 WT and *TAZ*^Δ45^ cells were treated with 20 μM CCCP for serial time points from 0 to 120 min. Whole cell lysates (45 μg) of the indicated lines and treatment times were immunoblotted for the indicated proteins. Densitometry quantification can be found in Figure S5. *H,* band intensities, relative to the loading control GRP75, for both full-length and cleaved PGAM5 were individually quantified and plotted as the percent of cleaved PGAM5 (cleaved/full + cleaved); WT 0 min n = 41, WT 10 min n = 8, WT 30 min n = 8, WT 60 min n = 8, WT 90 min n = 5, WT 120 min n = 7, *TAZ*^Δ45^ 0 min n = 41, *TAZ*^Δ45^ 10 min n = 8, *TAZ*^Δ45^ 30 min n = 7, *TAZ*^Δ45^ 60 min n = 5, *TAZ*^Δ45^ 90 min n = 7, and *TAZ*^Δ45^ 120 min n = 7. *I,* relative mRNA expression of *PGAM5* determined by qRT-PCR and ΔΔC_T_ quantification; WT 0 min n = 14, *TAZ*^Δ45^ 0 min n = 16, WT 120 min n = 16, and *TAZ*^Δ45^ 120 min n = 16. *J,* whole cell lysates (45 μg) of the indicated lines were immunoblotted for the indicated proteins. Band intensities, relative to the loading control GRP75, for both full-length and cleaved PGAM5 were individually quantified, and the percent of cleaved PGAM5 (cleaved/full + cleaved) is indicated for each lane (n = 1). Significant differences are indicated; ∗≤0.05, ∗∗≤0.005, ∗∗∗≤0.0005, and ∗∗∗∗≤0.00005. Each immunoblot image is a representative image of multiple biological replicates. Each (n) value indicates biological replicates performed on separate blots. CCCP, carbonyl cyanide *m*-chlorophenyl hydrazine; GRP75, glucose-regulated protein 75; HEK293, human embryonic kidney 293 cells; PARL, presenilin-associated rhomboid-like protein; PGAM5, phosphoglycerate mutase 5; TAZ, TAFFAZIN.

We next studied a potentially upregulated mitochondrial pathway in tafazzin-deficient cells involving PARL. Immunoblotting of WT and *TAZ*^Δ45^ HEK293 whole cell lysates confirmed the proteomics finding of increased PARL abundance (FC = 1.815; *p* = 0.016) (Fig. 3, *A* and *B*). There was also a subtle but significant increase in the relative mRNA expression of *PARL* in *TAZ*^Δ45^ cells (*p* = 0.02) (Fig. 3*C*). Thus, increased PARL in tafazzin-deficient HEK293 cells may be regulated both at the transcriptional and protein expression levels.

To investigate the biological significance of increased PARL abundance in *TAZ*^Δ45^ cells, we assessed a downstream proteolytic target of PARL, PGAM5. Previous evidence suggests that PGAM5 is cleaved by PARL and another stress-activated IMM protease, OMA1, in response to loss of mitochondrial membrane potential (ΔΨ_*m*_) (35). At baseline, we observed a significant increase in the percent of cleaved PGAM5 in *TAZ*^Δ45^ cells (*p* = 1.5 × 10^−7^) (Fig. 3, *D* and *H*). Importantly, PGAM5 cleavage was absent in PARL-deficient HEK293 cells at baseline demonstrating that in unstressed HEK293 cells, PGAM5 cleavage is primarily mediated by PARL (Fig. 3*J*).

To induce stress, cells were exposed to the membrane depolarizer carbonyl cyanide *m*-chlorophenyl hydrazine (CCCP) for increasing periods. With the exception of the final 120 min time point, *TAZ*^Δ45^ cells maintained a significantly greater abundance of PARL than WT (Fig. 3, *E* and *F* and Table S4). While CCCP treatment stimulated PGAM5 cleavage in both WT and *TAZ*^Δ45^ cells, the increased abundance of PARL in the latter correlated with an increased percentage of cleaved PGAM5 at all time points in *TAZ*^Δ45^ cells (Fig. 3, *G* and *H* and Table S5). The difference in the percentage of cleaved PGAM5 between WT and *TAZ*^Δ45^ cells increased over time, from an 11% difference at 0 min to an 18% difference at 120 min (Fig. 3, *G* and *H*). Immunoblotting showed that the increased percent of cleaved PGAM5 in *TAZ*^Δ45^ cells was largely because of reduction in full-length PGAM5 (Fig. 3*G*). To further demonstrate differences in PGAM5 cleavage between WT and *TAZ*^Δ45^ cells in response to depolarization, we examined PGAM5 cleavage with increasing CCCP concentrations ranging from 0 to 100 μM. We found that exposure to increasing concentrations of CCCP resulted in increased cleavage of PGAM5 in both WT and *TAZ*^Δ45^, but this cleavage was significantly greater in *TAZ*^Δ45^ cells compared with WT at all concentrations tested (Fig. S7). Importantly, PGAM5 cleavage was reduced in CCCP-treated PARL-KO HEK293 cells demonstrating that PGAM5 is predominantly cleaved by PARL under stress (Fig. 3*J*).

*PGAM5* mRNA expression was not significantly different between WT and *TAZ*^Δ45^ cells at 0 min or 120 min (Fig. 3*I*). There was a subtle but significant reduction in *PGAM5* mRNA expression between WT at 0 min and WT following 120 min CCCP treatment (Fig. 3*I*). Combined with the predicted negative impact of CCCP on import and assembly of mitochondrial precursors, this strongly suggests that the increased cleavage of PGAM5 in *TAZ*^Δ45^ cells at baseline and following CCCP treatment is due to the increased cleavage and degradation of existing full-length PGAM5.

In summary, we demonstrated baseline increased PARL abundance and PGAM5 cleavage, which is exacerbated upon mitochondrial depolarization in *tafazzin*-deficient HEK293 cells. We also showed that in PARL-KO HEK293 cells, PGAM5 cleavage at baseline and in the setting of inner membrane depolarization is severely reduced. Together with prior published *in vitro* data showing that overexpression of PARL results in increased PGAM5 cleavage with CCCP treatment (35), our data demonstrate that PGAM5 cleavage is primarily attributable to cleavage by PARL in this setting.

### Bromoenol lactone and SS-31 normalize cellular dysfunction in TAZ deficiency

To determine if targeting CL and CL metabolism would remediate decreased CI expression and/or increased PARL expression and PGAM5 cleavage, *TAZ*^Δ45^ and WT cells were treated with either bromoenol lactone (BEL) or SS-31. Previous studies have established that treatment with BEL, an inhibitor of calcium-independent PLA_2_, partially remediates CL abnormalities by reducing MLCL accumulation and CL depletion (36, 37). SS-31, a cell permeable mitochondria-targeted tetrapeptide, selectively binds CL, where it has been shown to stabilize cristae morphology, preserve mitochondrial bioenergetics, and potentially facilitate interactions between CL and specific CL-interacting proteins (38, 39, 40).

**Figure 4:**
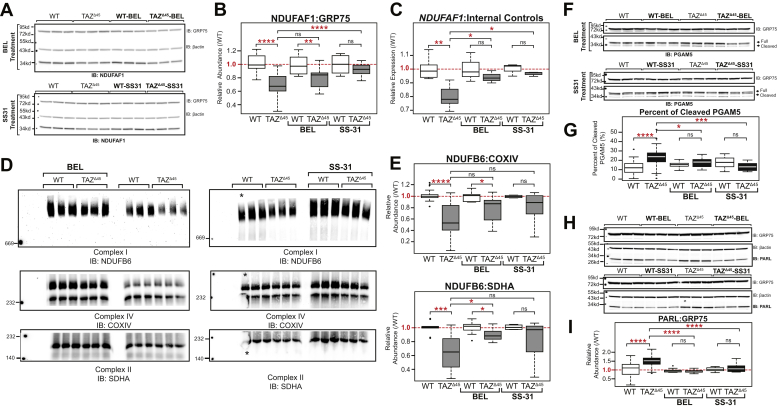
**Compounds that bind CL (SS-31) or inhibit CL deacylation (BEL) improve mitochondrial function intafazzin-deficient cells.***A,* HEK293 WT and *TAZ*^Δ45^ cells were treated for 48 h with 2.5 μM BEL and 7 days with 100 nM SS-31. Whole cell lysates (40–45 μg) of the indicated lines were immunoblotted for the indicated proteins. *B,* band intensities, relative to the loading control GRP75, were quantified and plotted relative to WT abundance; WT n = 27, *TAZ*^Δ45^ n = 26, WT-BEL n = 9, *TAZ*^Δ45^-BEL n = 9, WT-SS-31 n = 9, and *TAZ*^Δ45^-SS-31 n = 9. *C,* relative mRNA expression of *NDUFAF1* determined by qRT-PCR and ΔΔC_T_ quantification using each respective control; WT n = 6, *TAZ*^Δ45^ n = 6, WT-BEL n = 3, *TAZ*^Δ45^-BEL n = 3, WT-SS-31 n = 3, and *TAZ*^Δ45^-SS-31 n = 3 per gene. *D,* BN-PAGE of HEK293 WT and *TAZ*^Δ45^ cells treated for 48 h with 2.5 μM BEL (120 μg total protein) and 7 days with 100 nM SS-31 solubilized in 1% Triton X-100 immunoblotted for the indicated proteins. All samples were resolved on a single gel and exposed for the same duration. The WT lane indicated with the *asterisk* was not used for quantification because of air bubbles. *E,* band intensities were quantified, and CI abundance was represented as the ratio of CI band intensity to CIV or CII. Abundance was plotted relative to respective control; CI:CIV(WT n = 13, *TAZ*^Δ45^ n = 16, WT-BEL n = 9, *TAZ*^Δ45^-BEL n = 11, WT-SS-31 n = 4, and *TAZ*^Δ45^-SS-31 n = 6), CI:CII (WT n = 10, *TAZ*^Δ45^ n = 13, WT-BEL n = 9, *TAZ*^Δ45^-BEL n = 10, WT-SS-31 n = 4, and *TAZ*^Δ45^-SS-31 n = 6). *F,* whole cell lysates (40–45 μg) of the indicated lines and treatment conditions were immunoblotted for the indicated proteins. *G,* band intensities, relative to the loading control GRP75, for both full-length and cleaved PGAM5 were individually quantified and plotted as the percent of cleaved PGAM5 (cleaved/full + cleaved); WT n = 41, *TAZ*^Δ45^ n = 41, WT-BEL n = 16, *TAZ*^Δ45^-BEL n = 16, WT-SS-31 n = 9, and *TAZ*^Δ45^-SS-31 n = 8. *H,* whole cell lysate (40–45 μg) of the indicated lines and treatment conditions were immunoblotted for the indicated proteins. *I,* band intensities, relative to the loading control GRP75, were quantified and plotted relative to WT abundance; WT n = 54, *TAZ*^Δ45^ n = 48, WT-BEL n = 11, *TAZ*^Δ45^-BEL n = 11, WT-SS-31 n = 10, and *TAZ*^Δ45^-SS-31 n = 12. Significant differences are indicated; ∗≤0.05, ∗∗≤0.005, ∗∗∗≤0.0005, and ∗∗∗∗≤0.00005. BEL, bromoenol lactone; CI, complex I; CII, complex II; CIV, complex IV; CL, cardiolipin; GRP75, glucose-regulated protein 75; HEK293, human embryonic kidney 293; PGAM5, phosphoglycerate mutase 5; TAZ, TAFFAZIN.

The relative abundance of NDUFAF1 in *TAZ*^Δ45^ cells increased following BEL treatment and significantly increased following SS-31 treatment (*p* = 2.8 × 10^−5^) (Fig. 4, *A* and *B*). *NDUFAF1* relative mRNA expression significantly increased in *TAZ*^Δ45^ cells following both BEL and SS-31 treatment (*p* = 0.05 and *p* = 0.01, respectively) (Fig. 4*C*). The relative mRNA expression was also measured for the other four CI-associated genes that had significantly reduced levels in *TAZ*^Δ45^ cells at baseline (*NDUFB2*, *NDUFAB1*, *NDUFB4*, and *NDUFB6*). Following BEL treatment, these significant differences in expression between *TAZ*^Δ45^ *versus* WT were no longer observed in any of the four genes tested, and following SS-31 treatment, the significant differences were no longer observed in three of the four genes tested (Fig. S8).

CI holoenzyme abundance was measured in BEL and SS-31–treated cells by BN-PAGE. CI remained the most significantly reduced complex in *TAZ*^Δ45^ cells after BEL or SS-31 treatment (Fig. 4*D*). However, both treatments partially remediated the ratio of both CI to CIV (CI:CIV) and CI to CII (CI:CII) (Fig. 4, *D* and *E*). The CI:CIV relative abundance increased from 0.59 in untreated *TAZ*^Δ45^ cells to 0.77 in *TAZ*^Δ45^-BEL cells and 0.80 in *TAZ*^Δ45^-SS-31 cells (Fig. 4*E*). The CI:CII relative abundance significantly increased from 0.66 in untreated *TAZ*^Δ45^ cells to 0.89 in *TAZ*^Δ45^-BEL cells (*p* = 6.1 × 10^−3^) and to 0.83 in *TAZ*^Δ45^-SS-31 cells (not significant) (Fig. 4*E*). Overall, treatment with either BEL or SS-31 showed an increase in CI holoenzyme abundance.

The percentage of cleaved PGAM5 was significantly decreased in *TAZ*^Δ45^-BEL (18%; *p* = 0.01) and *TAZ*^Δ45^-SS-31 (13%; *p* = 2.6 × 10^−4^) cells compared with TAZ^Δ45^-untreated cells (23%) (Fig. 4, *F* and *G*). Moreover, there was no significant difference in the percentage of cleaved PGAM5 in *TAZ*^Δ45^-BEL *versus* WT-BEL cells or *TAZ*^Δ45^-SS-31 *versus* WT-SS-31 cells (Fig. 4*G*). Furthermore, PARL was significantly decreased in *TAZ*^Δ45^-BEL (FC = 0.93; *p* = 7.1 × 10^−15^) and *TAZ*^Δ45^-SS-31 (FC = 1.11; *p* = 9.9 × 10^−5^) cells compared with TAZ^Δ45^-untreated cells (FC = 1.53) (Fig. 4, *H* and *I*) and essentially restored to WT levels following each treatment. Collectively, these results indicate that compounds that bind CL or inhibit CL deacylation partially rescue the observed defects in CI and PARL–PGAM5 in tafazzin-deficient cells.

## Discussion

As a central phospholipid of the IMM, CL has been shown to have diverse roles in mitochondrial function (1). Yet, these diverse roles are currently underappreciated in the pathophysiology of BTHS, which is the only known Mendelian disorder of CL metabolism. In this study, we took an untargeted approach to identify dysregulated proteins in tafazzin-deficient HEK293 cells and pursued two areas of mitochondrial dysfunction for further study: decreased expression and function of CI of the respiratory chain and increased expression of the IMM rhomboid protease, PARL.

Mitochondrial respiratory chain dysfunction and supercomplex destabilization have been shown in several cellular models of BTHS, though a specific decrease of CI has only been described in a limited number of studies in patient-derived lymphoblasts (32, 33, 34). We show, for the first time, decreased gene and protein expression of specific subunits of CI. Furthermore, we show that expression of NDUFAF1, a CI assembly factor, is strikingly abnormal in the setting of tafazzin deficiency. We also demonstrated that decreased expression of CI gene subunits and assembly factors is partially rescued by drugs that target CL or its metabolism. As respiratory chain assembly and subunit gene and protein expression are tightly linked, it is not possible at this time to know which is the primary *versus* secondary in the setting of tafazzin deficiency (*i.e.*, whether decreased CL and/or increased MLCL affect one or all these CI-associated abnormalities), and this warrants further investigation (41). However, in demonstrating that CL modulation can in part rescue CI defects, we propose CI assembly, assembly factor expression (*NDUFAF1*), and subunit expression (*MT-ND3*, *NDUFA5*, *NDUFAB1*, *NDUFB2*, and *NDUFB4*) are valid cellular markers for treatment efficacy in BTHS.

Our untargeted proteomics approach in tafazzin-deficient cells also led us to identify increased expression of the IMM rhomboid protease PARL, which has roles in the mitochondrial response to stress *via* regulation of apoptosis and mitophagy through reciprocal cleavage of PGAM5 and phosphatase and tensin homolog–induced kinase 1 (42). PARL, and its cleavage of PGAM5, are of particular interest in BTHS, where enlarged mitochondria have been observed in BTHS patient and tafazzin-deficient mouse cardiac tissue, consistent with impaired mitophagy (43, 44, 45, 46). Similar to CI defects, targeting CL or its metabolism with BEL and SS-31 partially remediated PARL abundance and PGAM5 cleavage in the absence of tafazzin. The PARL proteolytic and signaling cascade has diverse downstream targets in addition to PGAM5, including phosphatase and tensin homolog–induced kinase 1, BCL-xL, FUNDC1, and others, with sundry effects on mitochondrial and cellular metabolism and survival, and thus represents a wide area for future study in tafazzin deficiency (47, 48).

It is also not clear at this time if the increased PARL expression and decreased CI expression/function are interdependent or are each independently initiated by IMM disturbances caused by CL abnormalities. One way to test this would be to correct the CI dysfunction in isolation and examine for remediation of PARL defects. Because overexpression of NDUFAF1 in the tafazzin-deficient cells was not sufficient to correct CI defects and test this hypothesis, other approaches such as downregulating PARL and assaying CI function/assembly could be considered.

Together, CI dysfunction and imbalances between apoptosis/mitophagy *via* PARL represent attractive areas for study in the tissue-specific aspects of BTHS. Defective MQC, particularly as it affects stress responsiveness, is increasingly recognized for its role in several forms of cardiac dysfunction (17), and both primary and secondary CI defects are well documented in many forms of cardiac dysfunction (18, 19). Furthermore, understanding the diverse mitochondrial effects caused by tafazzin deficiency has important implications for development of therapeutics, for example, CI targeting agents may not be sufficient to remediate cardiac or skeletal muscle abnormalities in BTHS, where underlying defects in mitophagy and apoptosis are prominent tafazzin-deficient–associated abnormalities.

## Conclusions

We identified and characterized two cellular pathways impacted by tafazzin deficiency by employing a discovery-based approach in a new HEK293 tafazzin-deficient cellular model: CI of the mitochondrial respiratory chain and the PARL–PGAM response to mitochondrial depolarization. We not only confirmed reduced CI protein and holoenzyme abundance but expanded our current understanding of respiratory chain abnormalities by showing reduced CI mRNA expression. In addition, we uncovered increased abundance of the inner membrane protease, PARL, which was accompanied by enhanced cleavage of its downstream target, PGAM5. Finally, we found that compounds that target CL or CL metabolism normalized CI gene expression, protein, and holoenzyme abundance, as well as ameliorated PARL abundance and PGAM5 cleavage when TAZ is missing.

## Experimental procedures

### Cell lines and culture conditions

HEK293 WT cells were purchased from American Type Culture Collection (293 (7)x ATCC CRL-1573). Cells were grown at 37 °C and 5% CO_2_. HEK293 WT and *TAZ*^Δ45^ were maintained in Dulbecco's modified Eagle's medium with l-glutamine and 4.5 g/l glucose (Corning Cellgro; catalog no. 10-017) containing 10% fetal bovine serum (Gemini) and 2 mM l-glutamine (Gibco; catalog no. 25030149). Mycoplasma contamination was routinely monitored and not detected.

For CCCP treatment, cells were seeded into 6-well plates. At confluence, cells were either treated with 20 μM CCCP (Sigma; catalog no. C2759) for 0, 10, 30, 60, 90, and 120 min or 0, 10, 20, 40, 80, and 100 μM CCCP for 45 min. For BEL treatment, cells (400 K) were seeded into 6-well plates. 48 h later, at 80 to 90% confluence, cells were treated with 2.5 μM BEL (Sigma; catalog no. B1552) for 48 h. For SS-31 treatment, cells (50 K) were seeded into 6-well plates. For 7 days, cells were fed fresh 100 nM SS-31 (provided by Stealth BioTherapeutics).

### CRISPR–Cas9 genome editing

sgRNAs (Fig. S1 and Table S1) targeting exon 2 of *TAFAZZIN* were designed at www.crispr.mit.edu and selected based on the scoring algorithm detailed by Hsu *et al*. (49, 50, 51) (Table S1). Synthesized sgRNA 1 and sgRNA 2 were individually cloned into pSpCas9(BB)-2A-Puro (PX459) V2.0 vector. pSpCas9(BB)-2A-Puro (PX459) V2.0 was a gift from Feng Zhang (Addgene plasmid no. 62988; http://n2t.net/addgene:62988; research resource identifier: Addgene_62988) (55). HEK293 WT cells were transfected with both sgRNA vectors using Lipofectamine 2000 (Invitrogen). About 24 h after transfection, cells were subjected to puromycin selection (2 μg/ml) for approximately 48 h. Following selection, cells were passaged in order to isolate single cell clones. Confluent colonies of single cell clones were collected using a cloning cylinder and expanded for DNA isolation and screening. For three *TAFAZZIN*-deficient clone isolates, we amplified and Sanger sequenced five of the top 10 predicted off-target sites, five off-target sites per sgRNA, which revealed no detectable off-target CRISPR–Cas9 genome editing activity (Table S1). The other five off-target sequences were not able to be amplified likely because of highly repetitive sequences and/or increased GC base pair content. None of the top 10 predicted off-target sites were located in coding regions. Still, in an effort to mitigate consequences of undetected off-target editing in any one of the clones, the clones, *TAZ*^Δ45.4^, *TAZ*^Δ45.5^, *TAZ*^Δ45.6^, were combined at a 1:1:1 ratio to create the cellular model *TAZ*^Δ45^.

### RT-PCR

RNA was extracted from an aliquot of 3 × 10^6^ cells using the RNeasy Plus Kit (Qiagen). Complementary DNA (cDNA) was synthesized from extracted RNA using the iScript cDNA Synthesis Kit (BioRad). The region surrounding the CRISPR–Cas9 target site was PCR amplified using AccuPrime GC-Rich DNA Polymerase (Invitrogen) (forward primer: 5′ TACATGAACCACCTGACCGT 3′ and reverse primer: 5′ CAGATGTGGCGGAGTTTCAG 3′). PCR products were resolved on a 0.8% agarose gel.

### Whole cell lysate extraction

An aliquot of 3 × 10^6^ cells was centrifuged for 4 min at 1000 rpm. The resultant cell pellet was washed twice with ice-cold PBS and lysed with radioimmunoprecipitation assay lysis buffer (1% [v/v] Triton X-100, 20 mm Hepes–KOH, pH 7.4, 50 mm NaCl, 1 mm EDTA, 2.5 mm MgCl_2_, and 0.1% [w/v] SDS) spiked with 1 mm PMSF for 30 min at 4 °C with rotation. Insoluble material was removed by centrifugation for 30 min at 21,000*g* at 4 °C, the supernatant collected, and protein quantified using a bicinchoninic acid assay (Pierce).

### Mitochondrial isolation

Mitochondrial isolation of HEK293 cells was performed as previously described by Lu *et al*. (2). Briefly, cells were suspended in PBS and centrifuged at 600*g* for 10 min. The pelleted cells were washed and resuspended in 3 ml isolation buffer (10 mm Tris–Mops, 1 mm EGTA–Tris, pH 7.4, and 200 mm sucrose) and homogenized. Cell lysates were centrifuged at 600*g* for 10 min two times to pellet the nuclear fraction. Mitochondria were then isolated from the supernatant by centrifugation at 7000*g* for 10 min. The mitochondrial pellet was resuspended and centrifuged again at 7000*g* spin and then 10,000*g* spin to obtain crude mitochondria.

### Immunoblotting

Whole cell extracts of mitochondria, resuspended in XT sample buffer (BioRad) and reducing agent (BioRad), were resolved on Criterion XT 12% Bis–Tris gels (BioRad) in XT Mops running buffer (BioRad). Proteins were transferred to Immuno-Blot polyvinylidene difluoride (BioRad) at 100 V for 1 h. Following transfer, membranes were blocked with 5% (w/v) milk and 0.05% (v/v) Tween-20 in PBS (blocking buffer) for 1 h at room temperature or at 4 °C if longer, with blocking. Following blocking, membranes were briefly washed with PBS with 0.2% (v/v) Tween-20 (PBST) and then incubated with primary antibody in PBST with 0.02% (w/v) sodium azide overnight at 4 °C with blocking. Following three successive 10-min washes with PBST at room temperature, horseradish peroxidase–conjugated secondary antibodies were added and incubated for 45 min at room temperature. The membranes were then washed three times for 10 min with PBST and twice for 10 min with PBS. Immunoreactive bands were visualized using ECL Western Blotting Detection (Amersham) or SuperSignal West Pico PLUS (Pierce). Images were captured with a Fluorchem Q (Cell Biosciences, Inc) or on film. Film was scanned before quantification. Quantitation of bands was performed using ImageJ (http://imagej.nih.gov/ij/) and protein expression values were normalized to loading controls.

### Antibodies

Antibodies against the following proteins were used; β-actin (loading control; Life Technologies; catalog no. AM4302), glucose-regulated protein 75 (loading control; Antibodies, Inc; catalog no. 75-127), TAZ (#2C2C9; Epitope: 237-TDFIQEEFQHL, exon 11) (3), NDUFAF1 (Abcam; catalog no. ab79826), PARL (Abcam; catalog no. ab45231; Proteintech; catalog no. 26679-1-AP; Langer Laboratory), ubiquinol–cyctochrome C reductase core protein 2 (Abcam: catalog no. ab14745), NDUFA9 (Abcam; catalog no. ab14713), NDUFS3 (Abcam; catalog no. ab110246), and NDUFB6 (Abcam: catalog no. ab110244). Two horseradish peroxidase–conjugated secondary antibodies were used; goat anti-rabbit (Abcam; catalog no. ab6721) and goat antimouse (Abcam; catalog no. ab205719).

### Lipidomics

Lipids were extracted from cell pellets (3 × 10^6^ cells) and analyzed as previously described by Houtkooper *et al*. (52).

### Proteomics

Samples (WT [n = 3] and *TAZ*^Δ45^ [n = 3], serial passages) were reduced in 5 mM DTT for 1 h at 56 °C, alkylated in 10 mM iodoacetamide for 30 min in the dark at room temperature, and precipitated in cold (−20 °C) acetone 10% (w/v) trichloroacetic acid. The precipitate was pelleted by centrifugation at 16,000*g* for 15 min, the supernatant was discarded, and the pellet was rinsed with cold acetone and dried at room temperature. The samples were reconstituted in 50 μl of 20% acetonitrile and 80 mM TEAB and digested with 3.3 μg of trypsin/Lys-C mix (Promega) at 37 °C overnight. The digested samples were labeled with tandem mass tag 10-plex reagent (Thermo; lot no. SK257889) and combined. The sample was lyophilized and reconstituted in 0.5 ml of 10 mM TEAB and fractionated by high (8, 9) pH-reversed phase chromatography into 84 fractions, which were concatenated into 24. Each of the 24 fractions was lyophilized and reconstituted in 2% acetonitrile and 0.1% formic acid and separated over a 90-min low (2, 3) pH-reversed phase gradient (120 min run time) for MS analysis on an Orbitrap Fusion.

MS data were acquired using serial data-dependent fragmentation of individual precursor ions (data-dependent acquisition). An intact precursor ion scan (MS1) spanning 400 to 1600 Th was acquired every 3 s at a resolution of 120,000 (at *m*/*z* = 200). Fragmentation scans (MS2) were acquired at 60,000 resolution between precursor scans by isolation of individual precursor ions at 0.6 Th resolution, accumulation to 5 × 10^4^ automatic gain control for a maximum injection time of 250 ms, and activation with beam collision at 38% energy. Mass accuracy was maintained throughout the experiment by internal calibrant lock mass.

Proteomics raw spectra were processed in Proteome Discoverer (version 2.2.0.38) using Mascot (version 2.5.1) against a complete SwissProt database, specifying the taxonomy *Homo sapiens* (taxonomy ID = 9606; release date v 2017-06-07; 20,206 sequences). The mass tolerances were set to 5 ppm and 0.01 Da for the precursor and fragment masses, respectively. Trypsin was selected as the protease, allowing two missed cleavages. Based on a decoy database search, peptide spectral matches were assigned a strict false discovery rate of 0.01, based on a *q* value validation. Only peptides that met these high criteria were selected for quantitation. Only unique peptides were used, and abundances were normalized on total peptide amount.

Protein quantification is based on the normalized median ratio of all spectra of tagged peptides from the same protein (53, 54). From analyzing technical replicates, we have assessed the accuracy and precision of relative quantitative experiments using isobaric mass tags in the proteomics core and have established that the technical variation contributes less than 10% to FCs (54). Protein ratios between cell types were directly calculated from the grouped protein abundances. An ANOVA-based hypothesis test was used to calculate the *p* values for the abundances of the individual proteins across the three biological replicates in each sample group.

### Functional annotation

We selected proteins with a protein abundance FC (*TAZ*^Δ45^/WT) less than or equal to 0.80 (FC ≤ 0.80) and proteins with an FC greater than or equal to 1.2 (FC ≥ 1.20). Each subset was individually uploaded to DAVID 6.8 and compared with the background “*Homo sapiens*” (18, 19). With the functional annotation tool, we pulled all KEGG pathways and GO terms for further analysis.

### Quantitative RT-PCR

Total RNA was extracted from an aliquot of 3 × 10^6^ cells using the RNeasy Plus Kit (Qiagen). cDNA was synthesized from extracted RNA using the iScript cDNA Synthesis Kit (BioRad) in 20 μl reactions according to the manufacturer's suggested protocol using 1 μg of RNA. cDNA was subsequently diluted 10-fold with water. About 2.4 μl of cDNA was analyzed in 12 μl reactions using the SsoAdvanced Universal SYBR Green Supermix (Bio-Rad) according to the manufacturer's instructions and included each respective forward and reverse gene-specific primers (Table S6). Each sample-primer reaction was plated in triplicate per plate. Each plate also included both no RT controls (no-RT) for each cDNA sample and no template controls (no-template) for each primer pair. The reaction conditions were as follows: 2 min at 95 °C, followed by 40 two-temperature cycles of 5 s at 95 °C and 30 s at 60 °C. For nuclear genes, expression of each gene was analyzed by the comparative CT (ΔΔCT) method with *TBP* and *HPRT1* being averaged as endogenous reference genes. For mitochondrial genes (*MT-ND3*), expression of each gene was analyzed by the comparative CT (ΔΔCT) method with *MT-RNR1*, *MT-CO1*, and *MT-ATP6* being averaged as endogenous reference genes. Values were represented as average FC relative to respective WT or untreated controls.

### 1D BN-PAGE

Cell pellets (100,000 cells, ∼120 μg) were solubilized for 30 min on ice in 20 mm Bis–Tris, 10% (v/v) glycerol, 50 mM NaCl, supplemented with 1% (v/v) Triton X-100, and protease inhibitors (PMSF, leupeptin, and pepstatin). Extracts were clarified by centrifugation for 30 min at 21,000*g* at 4 °C and analyzed by 1D BN/SDS-PAGE as described by Claypool *et al*. (55).

### CI and CII activity assays

The activity of CI was measured using the Complex I Enzyme Activity Microplate Assay Kit (Abcam; catalog no. ab109721) according to the manufacturer's instructions using 200 μg of isolated mitochondria from HEK293 cells. The activity of CII was measured using the Complex II Enzyme Activity Microplate Assay Kit (Abcam; catalog no. ab109908) according to the manufacturer's instructions using 200 μg of isolated mitochondria from HEK293 cells.

### NDUFAF1 transfection

HEK293 cells were seeded into 15 cm plates. When cells reached ∼80% confluency, cells were transiently transfected with *NDUFAF1* (NM_016013) C-Myc/DDK-tagged plasmid (Origene; catalog no. RC200029) with Lipofectamine 3000 (Thermo; catalog no. L3000001) according to manufacturer's instructions. About 75.4 μg of plasmid was transfected with 105 μl of Lipofectamine 3000 per 15 cm plate. The cells were grown in galactose media for 48 h before mitochondria extraction performed as described previously.

### Metabolomics

HEK cell samples undergoing metabolic analysis were initially kept on ice and washed with ice-cold PBS prior to collection, followed by centrifugation at 1500 rpm and 4 °C. Metabolites within the cell pellet were extracted with 80% HPLC-grade methanol (Fisher Scientific) and 20% MS-grade water as previously described (56). The extraction solution was then collected and evaporated using a speedvac and a lyophilizer, which resulted in a white powder of dried metabolites. The collected metabolites were subsequently resuspended in 50% (v/v) acetonitrile diluted with MS-grade water and analyzed *via* an Agilent 1260 HPLC and 6490 triple-quadrupole (QQQ) mass spectrometer.

The Agilent 1260 HPLC-autosampler system was kept at 4 °C for the entirety of the run to prevent any degradation within the samples. Optimal separation was achieved with a reverse-phase chromatography system with an aqueous mobile phase of MS-grade water with 0.1% formic acid and an organic mobile phase of 98% acetonitrile with 0.1% formic acid. The Discovery HS F5 HPLC Column (3 μm particle size, L × I.D. 15 cm × 2.1 mm; Sigma) with a compatible guard column (Sigma) was used and maintained at a temperature of 35 °C. The injection volume was 2 μl, and the runtime was 50 min per sample. The flow rate, buffer gradient, and mass spectrometer parameters for the method were the same as previously described (57).

Data from pure standards of each compound of interest were acquired prior and simultaneously with samples in identical setting: precursor and product ion transitions, collision energy, and ion polarity. Agilent MassHunter and Agilent Qualitative and Quantitative Analysis Software packages were used to analyze the metabolic profiles. The metabolite peaks were integrated for raw intensities and then normalized by protein concentration collected from the original cell pellet. Protein concentration was determined using a FilterMax F5 microplate reader and a bovine serum albumin standard.

### Data analysis

All statistical analyses were performed using R, version 3.3.2 (October 31, 2016) (58). Between-group comparisons were performed using Welch’s two-sample *t* test. Outliers, outside 1.5× the interquartile range above the upper quartile and below the lower quartile, were only removed for statistical analyses of the isogenic HEK293 cell lines.

## Data availability

The MS proteomics data have been deposited to the ProteomeXchange Consortium *via* the PRIDE partner repository with the dataset identifier PXD026731 and 10.6019/PXD026731. All remaining data are contained within the article and supporting information.

## Supporting information

This article contains supporting information.

## Conflict of interest

H. J. V. has received research support from Stealth BioTherapeutics. All other authors declare that they have no conflicts of interest with the contents of this article.

## References

[1] Anzmann A.F., Claypool S.M., Vernon H., Ahmad S.I. (2019). Handbook of Mitochondrial Dysfunction.

[2] Lu Y.W., Galbraith L., Herndon J.D., Lu Y.L., Pras-Raves M., Vervaart M., Van Kampen A., Luyf A., Koehler C.M., McCaffery J.M., Gottlieb E., Vaz F.M., Claypool S.M. (2016). Defining functional classes of Barth syndrome mutation in humans. Hum. Mol. Genet..

[3] Gonzalez I.L. (2005). Barth syndrome: TAZ gene mutations, mRNAs, and evolution. Am. J. Med. Genet. A..

[4] Xu Y., Malhotra A., Ren M., Schlame M. (2006). The enzymatic function of tafazzin. J. Biol. Chem..

[5] Zhang M., Mileykovskaya E., Dowhan W. (2002). Gluing the respiratory chain together. Cardiolipin is required for supercomplex formation in the inner mitochondrial membrane. J. Biol. Chem..

[6] Claypool S.M. (2009). Cardiolipin, a critical determinant of mitochondrial carrier protein assembly and function. Biochim. Biophys. Acta.

[7] Chicco A.J., Sparagna G.C. (2007). Role of cardiolipin alterations in mitochondrial dysfunction and disease. Am. J. Physiol. Cell Physiol.

[8] Kulik W., van Lenthe H., Stet F.S., Houtkooper R.H., Kemp H., Stone J.E., Steward C.G., Wanders R.J., Vaz F.M. (2008). Bloodspot assay using HPLC-tandem mass spectrometry for detection of Barth syndrome. Clin. Chem..

[9] Ferreira C., Thompson R., Vernon H., Adam M.P., Ardinger H.H., Pagon R.A., Wallace S.E., Bean L.J.H., Stephens K., Amemiya A. (1993). GeneReviews((R)).

[10] Barth P.G., Scholte H.R., Berden J.A., Van der Klei-Van Moorsel J.M., Luyt-Houwen I.E., Van 't Veer-Korthof E.T., Van der Harten J.J., Sobotka-Plojhar M.A. (1983). An X-linked mitochondrial disease affecting cardiac muscle, skeletal muscle and neutrophil leucocytes. J. Neurol. Sci..

[11] Roberts A.E., Nixon C., Steward C.G., Gauvreau K., Maisenbacher M., Fletcher M., Geva J., Byrne B.J., Spencer C.T. (2012). The Barth Syndrome Registry: Distinguishing disease characteristics and growth data from a longitudinal study. Am. J. Med. Genet. A..

[12] Thompson W.R., DeCroes B., McClellan R., Rubens J., Vaz F.M., Kristaponis K., Avramopoulos D., Vernon H.J. (2016). New targets for monitoring and therapy in Barth syndrome. Genet. Med..

[13] Claypool S.M., Koehler C.M. (2012). The complexity of cardiolipin in health and disease. Trends Biochem. Sci..

[14] Lesnefsky E.J., Chen Q., Hoppel C.L. (2016). Mitochondrial metabolism in aging heart. Circ. Res..

[15] Cole L.K., Mejia E.M., Vandel M., Sparagna G.C., Claypool S.M., Dyck-Chan L., Klein J., Hatch G.M. (2016). Impaired cardiolipin biosynthesis prevents hepatic steatosis and diet-induced obesity. Diabetes.

[16] Younossi Z., Anstee Q.M., Marietti M., Hardy T., Henry L., Eslam M., George J., Bugianesi E. (2018). Global burden of NAFLD and NASH: Trends, predictions, risk factors and prevention. Nat. Rev. Gastroenterol. Hepatol..

[17] Dorn G.W. (2015). Mitochondrial dynamism and heart disease: Changing shape and shaping change. EMBO Mol. Med..

[18] Scheubel R.J., Tostlebe M., Simm A., Rohrbach S., Prondzinsky R., Gellerich F.N., Silber R.E., Holtz J. (2002). Dysfunction of mitochondrial respiratory chain complex I in human failing myocardium is not due to disturbed mitochondrial gene expression. J. Am. Coll. Cardiol..

[19] Fassone E., Taanman J.W., Hargreaves I.P., Sebire N.J., Cleary M.A., Burch M., Rahman S. (2011). Mutations in the mitochondrial complex I assembly factor NDUFAF1 cause fatal infantile hypertrophic cardiomyopathy. J. Med. Genet..

[20] Guerrero-Castillo S., Baertling F., Kownatzki D., Wessels H.J., Arnold S., Brandt U., Nijtmans L. (2017). The assembly pathway of mitochondrial respiratory chain complex I. Cell Metab.

[21] Stiburek L., Fornuskova D., Wenchich L., Pejznochova M., Hansikova H., Zeman J. (2007). Knockdown of human Oxa1l impairs the biogenesis of F1Fo-ATP synthase and NADH:ubiquinone oxidoreductase. J. Mol. Biol..

[22] Potting C., Tatsuta T., Konig T., Haag M., Wai T., Aaltonen M.J., Langer T. (2013). TRIAP1/PRELI complexes prevent apoptosis by mediating intramitochondrial transport of phosphatidic acid. Cell Metab.

[23] Miliara X., Tatsuta T., Berry J.L., Rouse S.L., Solak K., Chorev D.S., Wu D., Robinson C.V., Matthews S., Langer T. (2019). Structural determinants of lipid specificity within Ups/PRELI lipid transfer proteins. Nat. Commun..

[24] Galluzzi L., Lopez-Soto A., Kumar S., Kroemer G. (2016). Caspases connect cell-death signaling to organismal homeostasis. Immunity.

[25] Spinazzi M., De Strooper B. (2016). Parl: The mitochondrial rhomboid protease. Semin. Cell Dev Biol.

[26] Shi G., McQuibban G.A. (2017). The mitochondrial rhomboid protease PARL is regulated by PDK2 to integrate mitochondrial quality control and metabolism. Cell Rep.

[27] Wai T., Saita S., Nolte H., Muller S., Konig T., Richter-Dennerlein R., Sprenger H.G., Madrenas J., Muhlmeister M., Brandt U., Kruger M., Langer T. (2016). The membrane scaffold SLP2 anchors a proteolytic hub in mitochondria containing PARL and the i-AAA protease YME1L. EMBO Rep..

[28] Yang C., Liu X., Yang F., Zhang W., Chen Z., Yan D., You Q., Wu X. (2017). Mitochondrial phosphatase PGAM5 regulates Keap1-mediated Bcl-xL degradation and controls cardiomyocyte apoptosis driven by myocardial ischemia/reperfusion injury. In Vitro Cell Dev Biol Anim.

[29] Wang B., Nie J., Wu L., Hu Y., Wen Z., Dong L., Zou M.H., Chen C., Wang D.W. (2018). AMPKalpha2 protects against the development of heart failure by enhancing mitophagy via PINK1 phosphorylation. Circ. Res..

[30] Shires S.E., Gustafsson A.B. (2018). Regulating renewable energy: Connecting AMPKalpha2 to PINK1/parkin-mediated mitophagy in the heart. Circ. Res..

[31] Vogel R.O., Janssen R.J., Ugalde C., Grovenstein M., Huijbens R.J., Visch H.J., van den Heuvel L.P., Willems P.H., Zeviani M., Smeitink J.A., Nijtmans L.G. (2005). Human mitochondrial complex I assembly is mediated by NDUFAF1. FEBS J..

[32] Chatzispyrou I.A., Guerrero-Castillo S., Held N.M., Ruiter J.P.N., Denis S.W., L I.J., Wanders R.J., van Weeghel M., Ferdinandusse S., Vaz F.M., Brandt U., Houtkooper R.H. (2018). Barth syndrome cells display widespread remodeling of mitochondrial complexes without affecting metabolic flux distribution. Biochim. Biophys. Acta Mol. Basis Dis..

[33] Gonzalvez F., D'Aurelio M., Boutant M., Moustapha A., Puech J.P., Landes T., Arnaune-Pelloquin L., Vial G., Taleux N., Slomianny C., Wanders R.J., Houtkooper R.H., Bellenguer P., Moller I.M., Gottlieb E. (2013). Barth syndrome: Cellular compensation of mitochondrial dysfunction and apoptosis inhibition due to changes in cardiolipin remodeling linked to tafazzin (TAZ) gene mutation. Biochim. Biophys. Acta.

[34] McKenzie M., Lazarou M., Thorburn D.R., Ryan M.T. (2006). Mitochondrial respiratory chain supercomplexes are destabilized in Barth Syndrome patients. J. Mol. Biol..

[35] Sekine S., Kanamaru Y., Koike M., Nishihara A., Okada M., Kinoshita H., Kamiyama M., Maruyama J., Uchiyama Y., Ishihara N., Takeda K., Ichijo H. (2012). Rhomboid protease PARL mediates the mitochondrial membrane potential loss-induced cleavage of PGAM5. J. Biol. Chem..

[36] Malhotra A., Edelman-Novemsky I., Xu Y., Plesken H., Ma J., Schlame M., Ren M. (2009). Role of calcium-independent phospholipase A2 in the pathogenesis of Barth syndrome. Proc. Natl. Acad. Sci. U S A..

[37] Lu Y.W., Claypool S.M. (2015). Disorders of phospholipid metabolism: An emerging class of mitochondrial disease due to defects in nuclear genes. Front. Genet..

[38] Szeto H.H. (2014). First-in-class cardiolipin-protective compound as a therapeutic agent to restore mitochondrial bioenergetics. Br. J. Pharmacol..

[39] Birk A.V., Chao W.M., Bracken C., Warren J.D., Szeto H.H. (2014). Targeting mitochondrial cardiolipin and the cytochrome c/cardiolipin complex to promote electron transport and optimize mitochondrial ATP synthesis. Br. J. Pharmacol..

[40] Chavez J.D., Tang X., Campbell M.D., Reyes G., Kramer P.A., Stuppard R., Keller A., Zhang H., Rabinovitch P.S., Marcinek D.J., Bruce J.E. (2020). Mitochondrial protein interaction landscape of SS-31. Proc. Natl. Acad. Sci. U S A..

[41] Tang J.X., Thompson K., Taylor R.W., Olahova M. (2020). Mitochondrial OXPHOS biogenesis: Co-regulation of protein synthesis, import, and assembly pathways. Int. J. Mol. Sci..

[42] Lysyk L., Brassard R., Touret N., Lemieux M.J. (2020). PARL protease: A glimpse at intramembrane proteolysis in the inner mitochondrial membrane. J. Mol. Biol..

[43] Clarke S.L., Bowron A., Gonzalez I.L., Groves S.J., Newbury-Ecob R., Clayton N., Martin R.P., Tsai-Goodman B., Garratt V., Ashworth M., Bowen V.M., McCurdy K.R., Damin M.K., Spencer C.T., Toth M.J. (2013). Barth syndrome. Orphanet J. Rare Dis..

[44] Lavandero S., Chiong M., Rothermel B.A., Hill J.A. (2015). Autophagy in cardiovascular biology. J. Clin. Invest..

[45] Lee E., Koo Y., Ng A., Wei Y., Luby-Phelps K., Juraszek A., Xavier R.J., Cleaver O., Levine B., Amatruda J.F. (2014). Autophagy is essential for cardiac morphogenesis during vertebrate development. Autophagy.

[46] Saric A., Andreau K., Armand A.S., Moller I.M., Petit P.X. (2015). Barth syndrome: From mitochondrial dysfunctions associated with aberrant production of reactive oxygen species to pluripotent stem cell studies. Front. Genet..

[47] Ma K., Zhang Z., Chang R., Cheng H., Mu C., Zhao T., Chen L., Zhang C., Luo Q., Lin J., Zhu Y., Chen Q. (2020). Dynamic PGAM5 multimers dephosphorylate BCL-xL or FUNDC1 to regulate mitochondrial and cellular fate. Cell Death Differ.

[48] Wu W., Lin C., Wu K., Jiang L., Wang X., Li W., Zhuang H., Zhang X., Chen H., Li S., Yang Y., Lu Y., Wang J., Zhu R., Zhang L. (2016). FUNDC1 regulates mitochondrial dynamics at the ER-mitochondrial contact site under hypoxic conditions. EMBO J..

[49] Perez-Riverol Y., Csordas A., Bai J., Bernal-Llinares M., Hewapathirana S., Kundu D.J., Inuganti A., Griss J., Mayer G., Eisenacher M., Perez E., Uszkoreit J., Pfeuffer J., Sachsenberg T. (2019). The PRIDE database and related tools and resources in 2019: improving support for quantification data. Nucleic Acids Res..

[50] Hsu P.D., Scott D.A., Weinstein J.A., Ran F.A., Konermann S., Agarwala V., Li Y., Fine E.J., Wu X., Shalem O., Cradick T.J., Marraffini L.A., Bao G., Zhang F. (2013). DNA targeting specificity of RNA-guided Cas9 nucleases.. Nat. Biotechnol..

[51] Ran F.A., Hsu P.D., Wright J., Agarwala V., Scott D.A., Zhang F. (2013). Genome engineering using the CRISPR-Cas9 system.. Nat. Protoc..

[52] Houtkooper R.H., Rodenburg R.J., Thiels C., van Lenthe H., Stet F., Poll-The B.T., Stone J.E., Steward C.G., Wanders R.J., Smeitink J., Kulik W., Vaz F.M. (2009). Cardiolipin and monolysocardiolipin analysis in fibroblasts, lymphocytes, and tissues using high-performance liquid chromatography-mass spectrometry as a diagnostic test for Barth syndrome. Anal. Biochem..

[53] Kammers K., Cole R.N., Tiengwe C., Ruczinski I. (2015). Detecting significant changes in protein abundance. EuPA Open Proteom.

[54] Herbrich S.M., Cole R.N., West K.P., Schulze K., Yager J.D., Groopman J.D., Christian P., Wu L., O'Meally R.N., May D.H., McIntosh M.W., Ruczinski I. (2013). Statistical inference from multiple iTRAQ experiments without using common reference standards. J. Proteome Res..

[55] Claypool S.M., Oktay Y., Boontheung P., Loo J.A., Koehler C.M. (2008). Cardiolipin defines the interactome of the major ADP/ATP carrier protein of the mitochondrial inner membrane. J. Cell Biol.

[56] Elgogary A., Xu Q., Poore B., Alt J., Zimmermann S.C., Zhao L., Fu J., Chen B., Xia S., Liu Y., Neisser M., Nguyen C., Lee R., Park J.K., Reyes J. (2016). Combination therapy with BPTES nanoparticles and metformin targets the metabolic heterogeneity of pancreatic cancer. Proc. Natl. Acad. Sci. U S A..

[57] Nguyen T., Kirsch B.J., Asaka R., Nabi K., Quinones A., Tan J., Antonio M.J., Camelo F., Li T., Nguyen S., Hoang G., Nguyen K., Udupa S., Sazeides C., Shen Y.A. (2019). Uncovering the role of N-Acetyl-Aspartyl-Glutamate as a glutamate reservoir in cancer. Cell Rep.

[58] R Core Team (2013). R: A language and environment for statistical computing.

