# Supplementary material for: Diverse mitochondrial abnormalities in a new cellular model of TAFFAZZIN deficiency are remediated by cardiolipin-interacting small molecules
Source: J Biol Chem. 2021 Jul 24;297(3):101005. doi: 10.1016/j.jbc.2021.101005 (PMC8384898; doi:10.1016/j.jbc.2021.101005)
Supplement: Figures S1–S8 and Table S1–S6 [file mmc1.pdf]

Supplementary Information for:

Title: Diverse mitochondrial abnormalities in a new cellular model of TAFFAZZIN deficiency are remediated by CL-interacting small molecules

Authors: Arianna F. Anzmann[1], Olivia L. Sniezek[1], Alexandra Pado[1], Veronica Busa[1], Frédéric M. Vaz[2], Simion D. Kreimer[3], Robert N. Cole[3], Anne Le[4], Brian J. Kirsch[4], Steven M. Claypool[5], Hilary J. Vernon[1*]

Corresponding author at: Department of Genetic Medicine, Johns Hopkins University School of Medicine, 733 N Broadway, MRB 512 Baltimore, Maryland, USA. Email: hvernon1@jhmi.edu (H.J. Vernon)

This PDF file includes:

Figures S1 to S8

Tables S1 to S6

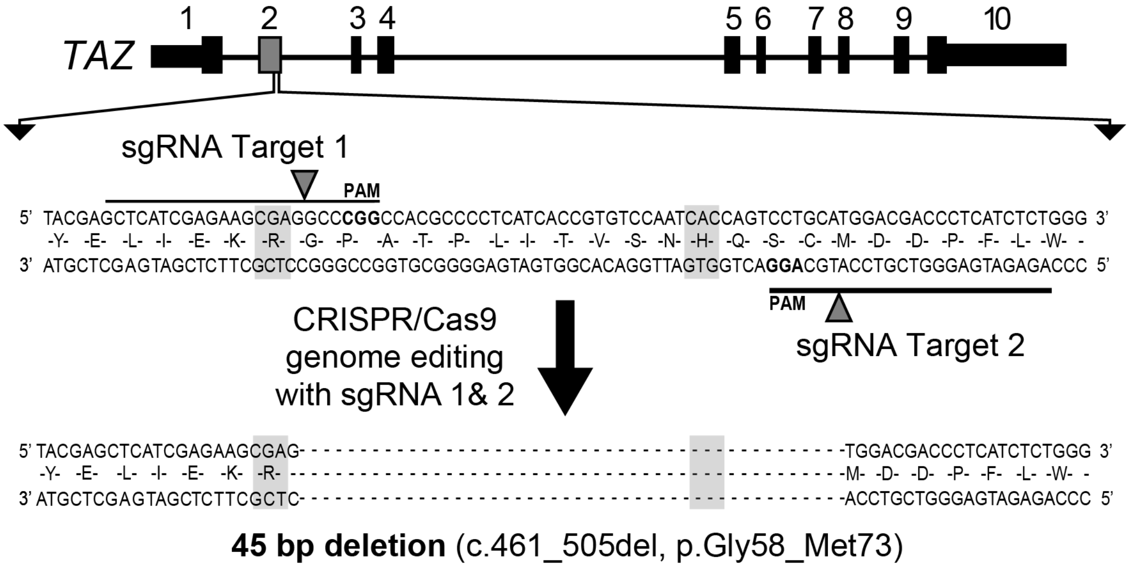

Figure S1. CRISPR/Cas9 genome editing with two single guide RNAs (sgRNAs) targeting exon 2 of *TAZ* results in a 45 bp deletion. The 45 bp deletion, noted by dashes, encompasses a predicted acyltransferase domain and covers an area of *TAZ* where multiple pathological variants have been described, such as p.R57L (c.170G>T) and p.H69Q (c.207C>G), highlighted in grey (3).

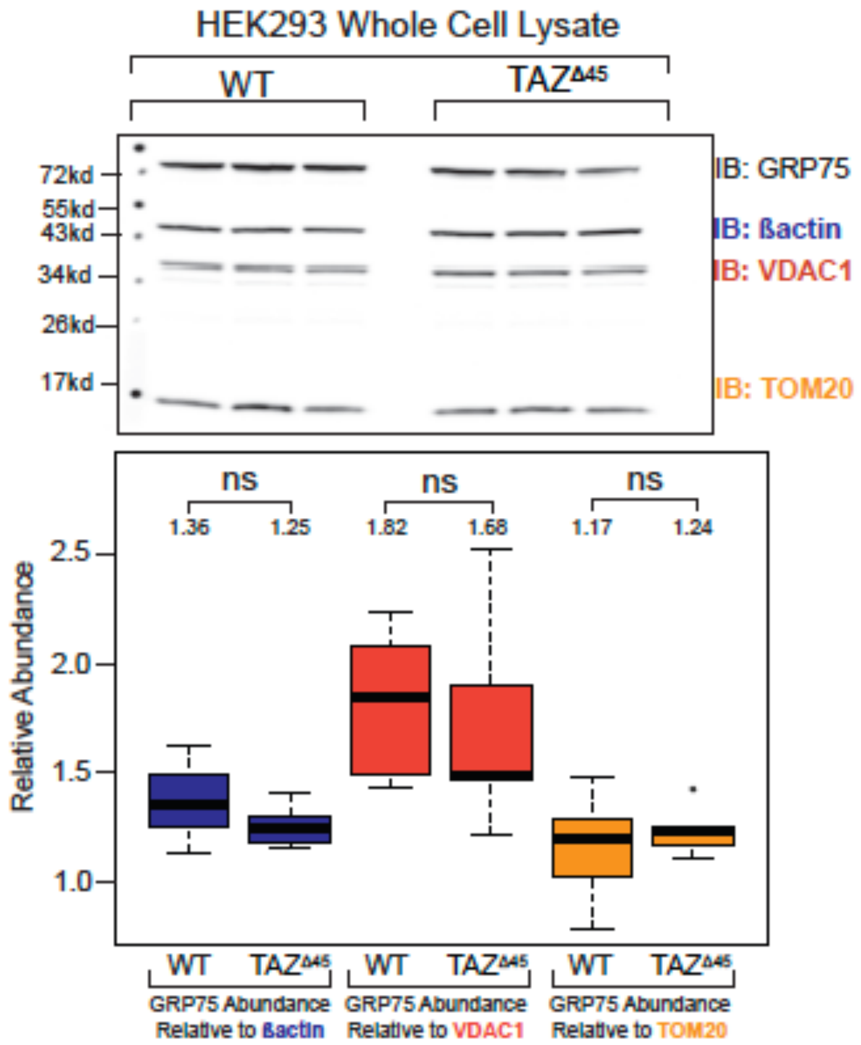

Figure S2. Comparable abundance of cytosolic and mitochondrial proteins in the absence of TAZ. Whole cell lysate (40 µg) of the indicated lines **(A)** HEK293 cells were immunoblotted for the indicated proteins. Band intensities were quantified and plotted relative to ß-actin (blue), VDAC1 (red), and TOM20 (yellow); WT n=9, *TAZ*$^{\Delta 45}$ n=9.

Table S1. CRISPR/Cas9 sgRNA guide sequences and predicted off-target sites.

sgRNA	Guide Sequence (5' to 3')	Off-Targets (5' to 3')	Score[1]	MMs[2]	Hg38 Location	Sequenced?
Target 1	GCTCATCGAGAAGCGAGGCC**CGG**[3]	GCCCGTCAAGAAGCGAGGCCCAG	2.4	3	chr9:-136416984	Y
		GCTCATTGTTAAGCGAGGCCTAG	0.9	3	chr5:-164071618	Y
		CTTCAGCCAGAAGCGAGGCCAAG	0.8	4	chr13:+111688858	Y
		CCCCATCGAGAAGCGCGGCCAAG	0.6	3	chr4:-132956077	N
		GCCCATCGGGAAGCCAGGCCGAG	0.5	3	chr18:-8629183	N
Target 2	GAGATGAGGGTCGTCCATGC**AGG**[3]	GAGATGTAGCTCGTCCATGCTGG	1.5	3	chr3:+173436340	Y
		GTTCAGAGGGTCGTCCATGCAAG	1.3	3	chr7:-70923313	Y
		GATATGAGGGGAGTCCATGCAGG	0.7	3	chrX:-12667869	N
		GACAAGAGGTTGGTCCATGCCAG	0.6	4	chr7:+65692780	N
		CTGGTGAGGGTCTTCCATGCCAG	0.6	4	chr8:-125971306	N

[1] Off-target score calculated at cripsr.mit.edu based on scoring algorithm from Hsu et al. 2013 (2)
[2] The number of mismatches between the guide sequence and the "off-target" sequence
[3] PAM site is bolded

Table S2A. Significant KEGG & GO terms determined by functional annotation analysis of the proteins with a FC ≤ 0.80 (n=215).

	# OF GENES[1]	%[2]	P-VALUE[3]	FOLD ENRICHMENT
**KEGG PATHWAYS**				
Parkinson's disease	13	6.1	6.80E-08	7.8
Oxidative phosphorylation*	11	5.1	2.70E-06	7
Huntington's disease	11	5.1	6.80E-05	4.9
Alzheimer's disease	10	4.7	1.30E-04	5.1
Non-alcoholic fatty liver disease (NAFLD)	9	4.2	3.40E-04	5.1
Cardiac muscle contraction	6	2.8	1.70E-03	6.8
Bile secretion	5	2.3	8.30E-03	6.2
Dilated cardiomyopathy	5	2.3	1.60E-02	5.1
Metabolic pathways*	23	10.7	1.90E-02	1.6
Ribosome	6	2.8	2.10E-02	3.7
**GO TERMS: BIOLOGICAL PROCESSES**				
platelet degranulation	10	4.7	2.60E-06	8.5
mitochondrial respiratory chain complex I assembly*#	7	3.3	7.90E-05	9.7
mitochondrial electron transport, NADH to ubiquinone*#	6	2.8	2.30E-04	10.7
actin filament organization	6	2.8	1.40E-03	7.3
response to oxidative stress	7	3.3	1.60E-03	5.6
protein lipoylation	3	1.4	2.60E-03	37.5
negative regulation of endothelial cell proliferation	4	1.9	4.30E-03	12.1
negative regulation of ATPase activity*	3	1.4	5.50E-03	26.2
aerobic respiration*	4	1.9	6.10E-03	10.6
protein targeting to mitochondrion*	4	1.9	6.70E-03	10.3
muscle contraction	6	2.8	7.50E-03	4.9
muscle filament sliding	4	1.9	9.10E-03	9.2
retina homeostasis	4	1.9	1.10E-02	8.7
rRNA processing	8	3.7	1.10E-02	3.3
wound healing	5	2.3	1.30E-02	5.5
Ossification	5	2.3	1.30E-02	5.5
cellular response to interferon-beta	3	1.4	1.40E-02	16.4
ribosomal small subunit biogenesis	3	1.4	1.40E-02	16.4
mitochondrial electron transport, cytochrome c to oxygen*	3	1.4	2.10E-02	13.1
SRP-dependent cotranslational protein targeting to membrane	5	2.3	2.20E-02	4.7
Translation	8	3.7	2.60E-02	2.8
cellular response to vascular endothelial growth factor stimulus	3	1.4	2.80E-02	11.4
response to calcium ion	4	1.9	2.80E-02	6

response to electrical stimulus	3	1.4	3.00E-02	10.9
positive regulation of osteoblast differentiation	4	1.9	3.10E-02	5.8
cytoplasmic translation	3	1.4	3.30E-02	10.5
ribosomal large subunit biogenesis	3	1.4	3.30E-02	10.5
viral transcription	5	2.3	3.90E-02	3.9
positive regulation of phagocytosis	3	1.4	4.30E-02	9
sarcomere organization	3	1.4	4.30E-02	9
one-carbon metabolic process*	3	1.4	4.50E-02	8.7
nuclear-transcribed mRNA catabolic process, nonsense-mediated decay	5	2.3	4.70E-02	3.7
vascular endothelial growth factor receptor signaling pathway	4	1.9	4.90E-02	4.9
**GO TERMS: CELLULAR COMPARTMENTS**				
mitochondrion*	42	19.6	2.30E-09	2.8
mitochondrial inner membrane*	20	9.3	5.80E-07	4
extracellular exosome	55	25.7	3.10E-05	1.7
prefoldin complex	4	1.9	4.70E-05	50.8
mitochondrial respiratory chain complex I*#	6	2.8	2.10E-04	10.9
platelet alpha granule lumen	6	2.8	3.70E-04	9.7
Cytosol	57	26.6	6.90E-04	1.5
Cytoskeleton	13	6.1	1.00E-03	3.1
cell surface	16	7.5	1.20E-03	2.6
focal adhesion	13	6.1	1.60E-03	3
mitochondrial proton-transporting ATP synthase complex*	4	1.9	1.60E-03	16.9
mitochondrial membrane*	6	2.8	4.10E-03	5.7
actin filament	5	2.3	6.10E-03	6.8
cytosolic large ribosomal subunit	5	2.3	7.10E-03	6.5
basement membrane	5	2.3	1.20E-02	5.6
Costamere	3	1.4	1.90E-02	14
integral component of mitochondrial inner membrane*	3	1.4	2.10E-02	13.3
respiratory chain*	3	1.4	2.10E-02	13.3
stress fiber	4	1.9	2.30E-02	6.6
Microvillus	4	1.9	2.60E-02	6.2
M band	3	1.4	2.70E-02	11.6
blood microparticle	6	2.8	2.80E-02	3.5
myelin sheath	6	2.8	2.80E-02	3.5
Cytoplasm	72	33.6	3.20E-02	1.2
Microspike	2	0.9	3.30E-02	59.3
granular component	2	0.9	4.40E-02	44.4
muscle thin filament tropomyosin	2	0.9	4.40E-02	44.4
apical plasma membrane	8	3.7	4.60E-02	2.4

filamentous actin	3	1.4	4.70E-02	8.6
extracellular matrix	8	3.7	4.90E-02	2.4
**GO TERMS: MOLECULAR FUNCTIONS**				
structural constituent of muscle	6	2.8	1.10E-04	12.6
protein binding	125	58.4	2.00E-04	1.3
actin filament binding	8	3.7	7.60E-04	5.3
unfolded protein binding	7	3.3	1.60E-03	5.6
NADH dehydrogenase (ubiquinone) activity *#	5	2.3	2.10E-03	9.2
poly(A) RNA binding	25	11.7	2.10E-03	1.9
structural constituent of ribosome	9	4.2	3.80E-03	3.6
endopeptidase inhibitor activity	4	1.9	1.00E-02	8.8
identical protein binding	17	7.9	1.10E-02	2
structural molecule activity conferring elasticity	2	0.9	2.30E-02	87.9
structural constituent of cytoskeleton	5	2.3	3.60E-02	4
ubiquitin conjugating enzyme activity	3	1.4	4.20E-02	9.1
cytochrome-c oxidase activity *	3	1.4	4.50E-02	8.8
* References mitochondrion and/or metabolic pathways (n=18)<br># References complex I of the oxidative phosphorylation pathways (n=4)				
[1] The number of input proteins involved in the term<br>[2] The number of input proteins involved in the term divided by the total proteins/genes represented by the term<br>[3] Modified Fisher Exact p-value, EASE Score				

Table S2B. Significant KEGG & GO terms determined by functional annotation analysis of the proteins with a FC ≥ 1.20 (n=621).

	# OF GENES[1]	%[2]	P-VALUE[3]	FOLD ENRICHMENT
**KEGG PATHWAYS**				
Chronic myeloid leukemia	9	1.4	2.50E-03	3.8
Metabolic pathways*	58	9.3	3.10E-03	1.4
Pancreatic cancer	8	1.3	5.40E-03	3.7
AMPK signaling pathway	11	1.8	7.50E-03	2.7
Alzheimer’s disease	13	2.1	9.70E-03	2.3
Butanoate metabolism*	5	0.8	1.10E-02	5.6
Carbohydrate digestion and absorption	6	1	1.20E-02	4.3
Endocytosis	16	2.6	1.40E-02	2
Insulin signaling pathway	11	1.8	1.60E-02	2.4
Colorectal cancer	7	1.1	1.60E-02	3.4
Fatty acid metabolism*	6	1	2.10E-02	3.8
Notch signaling pathway	6	1	2.10E-02	3.8

Amino sugar and nucleotide sugar metabolism*	6	1	2.10E-02	3.8
p53 signaling pathway	7	1.1	2.30E-02	3.1
Biosynthesis of antibiotics	14	2.3	2.30E-02	2
Phagosome	11	1.8	2.70E-02	2.2
Adipocytokine signaling pathway	7	1.1	2.80E-02	3
Inositol phosphate metabolism*	7	1.1	3.00E-02	3
Proteoglycans in cancer	13	2.1	3.30E-02	2
Biosynthesis of unsaturated fatty acids	4	0.6	3.90E-02	5.2
Viral myocarditis	6	1	4.00E-02	3.2
Synthesis and degradation of ketone bodies	3	0.5	4.10E-02	9
Phosphatidylinositol signaling system	8	1.3	4.30E-02	2.5
Glucagon signaling pathway	8	1.3	4.60E-02	2.4
Cell adhesion molecules (CAMs)	10	1.6	4.60E-02	2.1
Fatty acid elongation	4	0.6	4.80E-02	4.8
Epstein-Barr virus infection	9	1.4	4.90E-02	2.2
**GO TERMS: BIOLOGICAL PROCESSES**				
covalent chromatin modification	14	2.3	7.30E-05	3.8
viral genome replication	5	0.8	8.40E-04	11
protein targeting to plasma membrane	6	1	1.30E-03	7.1
antigen processing and presentation of endogenous peptide antigen via MHC class I via ER pathway, TAP-independent	3	0.5	3.10E-03	30.9
positive regulation of apoptotic process*	20	3.2	4.20E-03	2.1
carbohydrate phosphorylation	5	0.8	5.90E-03	6.7
cell cycle arrest	12	1.9	6.10E-03	2.6
response to oxidative stress*	10	1.6	9.30E-03	2.8
cell migration	13	2.1	1.00E-02	2.3
phosphatidylinositol biosynthetic process	7	1.1	1.10E-02	3.7
long-chain fatty-acyl-CoA biosynthetic process	6	1	1.10E-02	4.4
macroautophagy*	8	1.3	1.10E-02	3.2
unsaturated fatty acid biosynthetic process	4	0.6	1.10E-02	8.2
muscle cell differentiation	4	0.6	1.10E-02	8.2
IRE1-mediated unfolded protein response	7	1.1	1.20E-02	3.7
positive regulation of DNA binding	5	0.8	1.20E-02	5.5
tRNA pseudouridine synthesis	3	0.5	1.40E-02	15.4
magnesium ion homeostasis	3	0.5	1.40E-02	15.4
antigen processing and presentation of peptide antigen via MHC class I	5	0.8	1.50E-02	5.1
neuron projection development	9	1.4	1.60E-02	2.8
positive regulation of substrate adhesion-dependent cell spreading	5	0.8	1.90E-02	4.8
membrane protein intracellular domain proteolysis	4	0.6	1.90E-02	6.9
cilium assembly	10	1.6	1.90E-02	2.5

regulation of apoptotic process*	14	2.3	2.10E-02	2
inner ear receptor stereocilium organization	4	0.6	2.20E-02	6.5
regulation of autophagy*	6	1	2.20E-02	3.7
amyloid precursor protein catabolic process	3	0.5	2.60E-02	11.6
response to cholesterol	3	0.5	2.60E-02	11.6
interferon-gamma-mediated signaling pathway	7	1.1	2.70E-02	3
protein maturation by protein folding	3	0.5	3.20E-02	10.3
pyrimidine nucleotide metabolic process*	3	0.5	3.20E-02	10.3
DNA topological change	3	0.5	3.20E-02	10.3
antigen processing and presentation of exogenous peptide antigen via MHC class I, TAP-independent	3	0.5	3.20E-02	10.3
cilium morphogenesis	10	1.6	3.30E-02	2.3
cell growth	6	1	3.40E-02	3.3
viral process	17	2.7	3.40E-02	1.8
purine nucleotide metabolic process*	3	0.5	4.00E-02	9.3
regulation of synaptic vesicle exocytosis	3	0.5	4.00E-02	9.3
cell volume homeostasis	3	0.5	4.00E-02	9.3
nucleocytoplasmic transport	4	0.6	4.60E-02	4.9
positive regulation of stress fiber assembly*	5	0.8	4.60E-02	3.7
UDP-N-acetylglucosamine biosynthetic process	3	0.5	4.70E-02	8.4
positive regulation of histone deacetylation	3	0.5	4.70E-02	8.4
chromatin organization	5	0.8	4.90E-02	3.6
histone H3 acetylation	5	0.8	4.90E-02	3.6
glycosaminoglycan catabolic process	4	0.6	5.00E-02	4.7
**GO TERMS: CELLULAR COMPARTMENTS**				
extracellular exosome	135	21.7	1.70E-06	1.5
membrane	110	17.7	3.70E-06	1.5
endoplasmic reticulum	51	8.2	1.90E-05	1.9
mitochondrion*	68	11	2.20E-04	1.6
cytosol	142	22.9	2.80E-04	1.3
cytoplasm	206	33.2	7.60E-04	1.2
nucleoplasm	120	19.3	8.10E-04	1.3
integral component of endoplasmic reticulum membrane	11	1.8	2.00E-03	3.3
integral component of luminal side of endoplasmic reticulum membrane	6	1	2.20E-03	6.4
phagocytic vesicle membrane	8	1.3	2.90E-03	4.2
endoplasmic reticulum membrane	44	7.1	3.60E-03	1.6
intermediate filament	11	1.8	3.80E-03	3
ruffle membrane	9	1.4	5.10E-03	3.4
mitochondrial inner membrane*	26	4.2	5.20E-03	1.8
ER to Golgi transport vesicle membrane	7	1.1	6.60E-03	4.1

mitochondrial intermembrane space*	8	1.3	1.00E-02	3.3
myelin sheath	12	1.9	1.10E-02	2.4
nucleus	202	32.5	1.30E-02	1.1
melanosome	9	1.4	1.70E-02	2.7
early endosome	15	2.4	1.80E-02	2
Golgi membrane	30	4.8	1.80E-02	1.6
centrosome	23	3.7	2.20E-02	1.7
costamere	4	0.6	2.20E-02	6.5
endoplasmic reticulum-Golgi intermediate compartment	7	1.1	2.30E-02	3.2
endoplasmic reticulum lumen	13	2.1	2.30E-02	2.1
cilium	11	1.8	2.60E-02	2.2
autolysosome	3	0.5	2.60E-02	11.5
ER-mitochondrion membrane contact site*	3	0.5	2.60E-02	11.5
integral component of mitochondrial outer membrane*	4	0.6	2.90E-02	5.9
stress fiber*	6	1	3.00E-02	3.4
F-actin capping protein complex	3	0.5	3.30E-02	10.2
nuclear heterochromatin	4	0.6	3.30E-02	5.6
lysosomal membrane	16	2.6	3.50E-02	1.8
early endosome membrane	9	1.4	3.50E-02	2.4
Golgi apparatus	39	6.3	3.60E-02	1.4
oligosaccharyltransferase complex	3	0.5	4.00E-02	9.2
dendrite	18	2.9	4.70E-02	1.7
MHC class I protein complex	3	0.5	4.80E-02	8.4
**GO TERMS: MOLECULAR FUNCTIONS**				
protein binding	335	53.9	2.90E-05	1.2
poly(A) RNA binding	55	8.9	3.10E-03	1.5
AP-3 adaptor complex binding	3	0.5	3.10E-03	30.5
TAP binding	3	0.5	3.10E-03	30.5
nucleosomal DNA binding	7	1.1	3.70E-03	4.6
cysteine-type endopeptidase activity involved in apoptotic process*	4	0.6	7.80E-03	9.4
chromatin binding	23	3.7	9.80E-03	1.8
1-phosphatidylinositol-3-phosphate 4-kinase activity	3	0.5	1.00E-02	18.3
GTPase activity	16	2.6	1.00E-02	2.1
peptide antigen binding	5	0.8	1.20E-02	5.5
GTP binding	22	3.5	1.50E-02	1.7
histone binding	10	1.6	1.90E-02	2.5
scaffold protein binding	6	1	2.00E-02	3.8
1-phosphatidylinositol-4-phosphate 5-kinase activity	3	0.5	2.00E-02	13.1
chromatin DNA binding	6	1	4.10E-02	3.2

cysteine-type endopeptidase activity	6	1	4.90E-02	3
* References mitochondrion and/or mitochondrial dynamics (n=20)				

[1] The number of input proteins involved in the term
[2] The number of input proteins involved in the term divided by the total proteins/genes represented by the term
[3] Modified Fisher Exact p-value, EASE Score

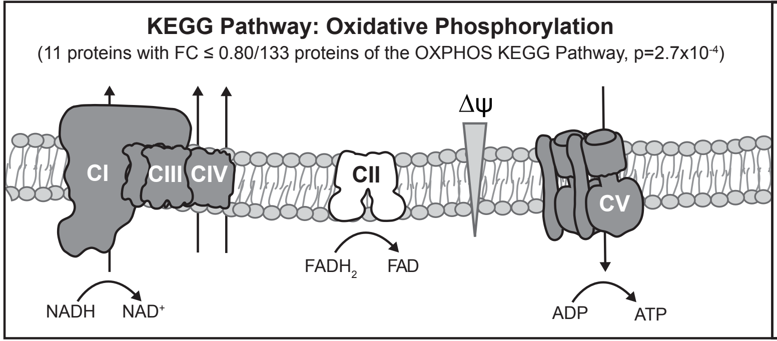

Oxidative Phosphorylation Complexes	
I	MT-ND3, NDUFA5, NDUFAB1, NDUFB2, NDUFB4
III	UQCRB
IV	COX1, COX6C, COX7C
V	ATP8, ATP5G3

Figure S3. The oxidative phosphorylation (OXPHOS) KEGG pathway is the most significant KEGG pathway enriched for proteins with a FC ≤ 0.80 that references mitochondria and or OXPHOS. Of the 133 genes in the OXPHOS KEGG pathway 11 encode proteins with a FC ≤ 0.80 in *TAZ*$^{\Delta 45}$ cells; half (n=5) are subunits of complex I (CI) and the remaining (n=6) are subunits of complex III, IV, and V.

Table S3. Proteomics quantification of all complex I (CI) associated proteins.

UniProt Entry	Gene Name	Module	Protein	#PSMs*	Unique Peptides	FC	P-Value
**Complex I Subunits**							
Q9UI09	*NDUFA12*	N	NADH dehydrogenase [ubiquinone] 1 alpha subcomplex subunit 12	46	8	0.973	0.93
P28331	*NDUFS1*	N	NADH-ubiquinone oxidoreductase 75 kDa subunit, mitochondrial	173	39	0.864	0.28
O75380	*NDUFS6*	N	NADH dehydrogenase [ubiquinone] iron-sulfur protein 6, mitochondrial	23	9	0.955	0.49
P49821	*NDUFV1*	N	NADH dehydrogenase [ubiquinone] flavoprotein 1, mitochondrial	105	21	0.872	0.21
P19404	*NDUFV2*	N	NADH dehydrogenase [ubiquinone] flavoprotein 2, mitochondrial	73	15	0.872	0.18
P56181	*NDUFV3*	N	NADH dehydrogenase [ubiquinone] flavoprotein 3, mitochondrial	5	3	1.037	0.69
O43678	*NDUFA2*	N/Q	NADH dehydrogenase [ubiquinone] 1 alpha subcomplex subunit 2	19	4	0.862	0.15
O43181	*NDUFS4*	N/Q	NADH dehydrogenase [ubiquinone] iron-sulfur protein 4, mitochondrial	21	7	0.890	0.22
**Q16718**	***NDUFA5***	**Q**	**NADH dehydrogenase [ubiquinone] 1 alpha subcomplex subunit 5**	**33**	**7**	**0.722**	**0.24**
P56556	*NDUFA6*	Q	NADH dehydrogenase [ubiquinone] 1 alpha subcomplex subunit 6	27	7	0.902	0.89
O95182	*NDUFA7*	Q	NADH dehydrogenase [ubiquinone] 1 alpha subcomplex subunit 7	33	9	0.834	0.21
Q16795	*NDUFA9*	Q	NADH dehydrogenase [ubiquinone] 1 alpha subcomplex subunit 9, mitochondrial	54	18	0.970	0.99
O75306	*NDUFS2*	Q	NADH dehydrogenase [ubiquinone] iron-sulfur protein 2, mitochondrial	130	22	1.018	0.95
O75489	*NDUFS3*	Q	NADH dehydrogenase [ubiquinone] iron-sulfur protein 3, mitochondrial	142	18	0.953	0.53
O75251	*NDUFS7*	Q	NADH dehydrogenase [ubiquinone] iron-sulfur protein 7, mitochondrial	27	6	0.873	0.94
O00217	*NDUFS8*	Q	NADH dehydrogenase [ubiquinone] iron-sulfur protein 8, mitochondrial	66	9	0.959	0.46
P03886	*MTND1*	P_P	NADH-ubiquinone oxidoreductase chain 1	4	2	1.236	0.43
P03891	*MTND2*	P_P	NADH-ubiquinone oxidoreductase chain 2	3	2	1.358	0.24
**P03897**	***MTND3****	**P_P**	**NADH-ubiquinone oxidoreductase chain 3**	**2**	**1**	**0.608**	**0.01**
P03923	*MTND6*	P_P	NADH-ubiquinone oxidoreductase chain 6	2	1	1.391	0.45
O95299	*NDUFA10*	P_P	NADH dehydrogenase [ubiquinone] 1 alpha subcomplex subunit 10, mitochondrial	56	17	0.957	0.82
Q86Y39	*NDUFA11*	P_P	NADH dehydrogenase [ubiquinone] 1 alpha subcomplex subunit 11	14	3	0.84	0.20
Q9P0J0	*NDUFA13*	P_P	NADH dehydrogenase [ubiquinone] 1 alpha subcomplex subunit 13	20	8	0.986	0.90
O95167	*NDUFA3*	P_P	NADH dehydrogenase [ubiquinone] 1 alpha subcomplex subunit 3	17	4	0.96	0.97
P51970	*NDUFA8*	P_P	NADH dehydrogenase [ubiquinone] 1 alpha subcomplex subunit 8	31	7	0.937	0.43
O95298	*NDUFC2*	P_P	NADH dehydrogenase [ubiquinone] 1 subunit C2	19	6	0.808	0.10
O43920	*NDUFS5*	P_P	NADH dehydrogenase [ubiquinone] iron-sulfur protein 5	29	7	0.935	0.22
P03905	*MTND4*	P_D	NADH-ubiquinone oxidoreductase chain 4	3	2	1.036	0.57
P03915	*MTND5*	P_D	NADH-ubiquinone oxidoreductase chain 5	11	3	0.977	1.00
**O14561**	***NDUFAB1***	**P_D**	**Acyl carrier protein, mitochondrial**	**6**	**2**	**0.727**	**0.46**
O75438	*NDUFB1*	P_D	NADH dehydrogenase [ubiquinone] 1 beta subcomplex subunit 1	3	2	0.995	0.98
O96000	*NDUFB10*	P_D	NADH dehydrogenase [ubiquinone] 1 beta subcomplex subunit 10	40	8	0.904	0.47
Q9NX14	*NDUFB11*	P_D	NADH dehydrogenase [ubiquinone] 1 beta subcomplex subunit 11, mitochondrial	20	3	0.863	0.10
**O95178**	***NDUFB2***	**P_D**	**NADH dehydrogenase [ubiquinone] 1 beta subcomplex subunit 2**	**6**	**1**	**0.62**	**0.12**
O43676	*NDUFB3***	P_D	NADH dehydrogenase [ubiquinone] 1 beta subcomplex subunit 3	11	3	0.864	0.05
**O95168**	***NDUFB4***	**P_D**	**NADH dehydrogenase [ubiquinone] 1 beta subcomplex subunit 4**	**30**	**4**	**0.762**	**0.22**
O43674	*NDUFB5*	P_D	NADH dehydrogenase [ubiquinone] 1 beta subcomplex subunit 5, mitochondrial	28	6	0.918	1.00
O95139	*NDUFB6*	P_D	NADH dehydrogenase [ubiquinone] 1 beta subcomplex subunit 6	18	7	1.081	0.99
P17568	*NDUFB7*	P_D	NADH dehydrogenase [ubiquinone] 1 beta subcomplex subunit 7	11	4	0.814	0.30
O95169	*NDUFB8*	P_D	NADH dehydrogenase [ubiquinone] 1 beta subcomplex subunit 8, mitochondrial	13	4	0.911	0.25
Q9Y6M9	*NDUFB9*	P_D	NADH dehydrogenase [ubiquinone] 1 beta subcomplex subunit 9	38	8	0.92	0.17
**Complex I Assembly Factors**							
Q9H845	*ACAD9*		Acyl-CoA dehydrogenase family member 9, mitochondrial	80	30	1.107	0.15
Q9GZY4	*COA1*		Cytochrome c oxidase assembly factor 1 homolog	6	4	1.119	0.78
Q9BQ95	*ECSIT*		Evolutionarily conserved signaling intermediate in Toll pathway, mitochondrial	12	9	0.918	0.47
Q96CU9	*FOXRED1*		FAD-dependent oxidoreductase domain-containing protein 1	13	6	0.96	0.97
**Q9Y375**	***NDUFAF1****		**Complex I intermediate-associated protein 30, mitochondrial**	**11**	**8**	**0.797**	**0.004**
Q8N183	*NDUFAF2*		NADH dehydrogenase [ubiquinone] 1 alpha subcomplex assembly factor 2	49	13	0.855	0.35
Q9BU61	*NDUFAF3*		NADH dehydrogenase [ubiquinone] 1 alpha subcomplex assembly factor 3	32	7	0.942	0.88
Q9P032	*NDUFAF4*		NADH dehydrogenase [ubiquinone] 1 alpha subcomplex assembly factor 4	30	10	0.952	0.30
Q5TEU4	*NDUFAF5*		Arginine-hydroxylase NDUFAF5, mitochondrial	1	1	0.98	0.73
Q330K2	*NDUFAF6*		NADH dehydrogenase (ubiquinone) complex I, assembly factor 6	1	1	0.998	0.86

Q7L592	*NDUFAF7*	Protein arginine methyltransferase NDUFAF7, mitochondrial	16	10	0.95	0.99
Q8TB37	*NUBPL*	Iron-sulfur protein NUBPL	7	5	0.952	1.00
Q9NPL8	*TIMMDC1*	Complex I assembly factor TIMMDC1, mitochondrial	23	8	1.118	0.02
Q8IUX1	*TMEM126B*	Complex I assembly factor TMEM126B, mitochondrial	1	1	0.984	0.96
Q9BUB7	*TMEM70*	Transmembrane protein 70, mitochondrial	5	3	1.063	0.84

[*] Peptide spectral matches
[Bold Font] Subunits or assembly factors with FC ≤ 0.80 (n=6)
[**] Significantly reduced subunits or assembly factors (n=2)

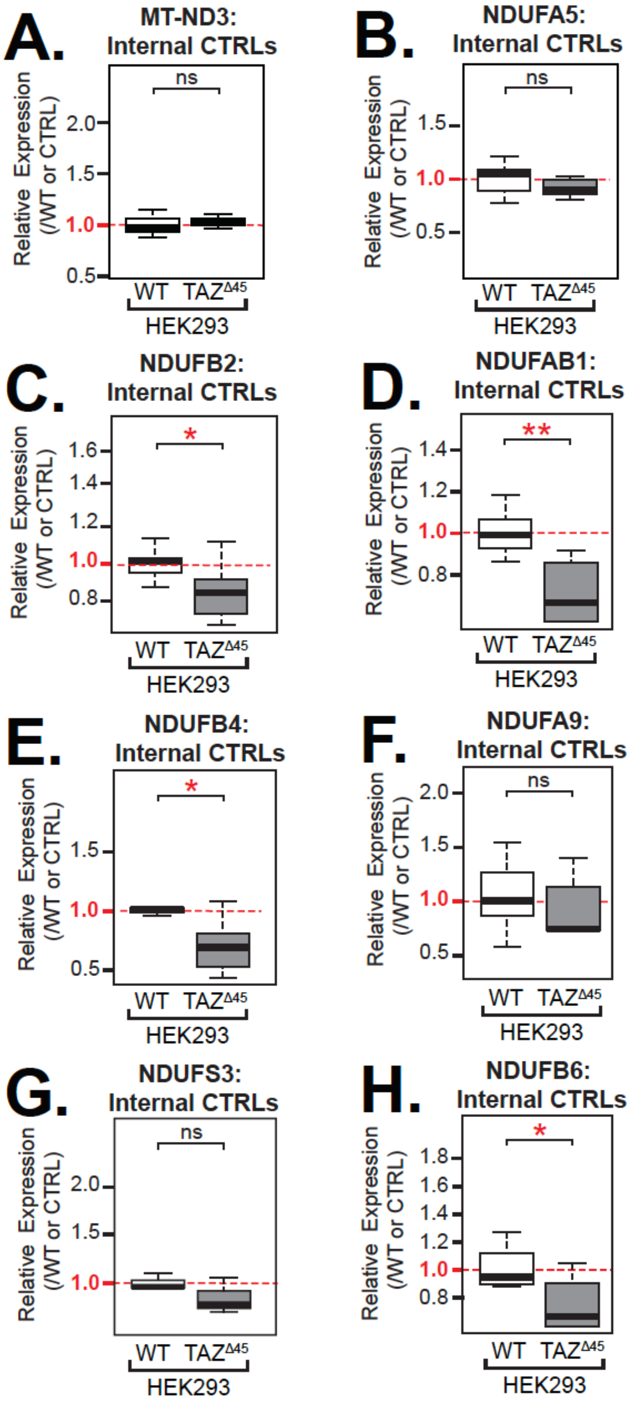

Figure S4. Relative mRNA expression of complex I (CI) subunits; (A) *MT-ND3* **(B)** *NDUFA5* **(C)** *NDUFB2* **(D)** *NDUFAB1* **(E)** *NDUFB4* **(F)** *NDUFA9* **(G)** *NDUFS3* **(H)** *NDUFB6* determined by qRT-PCR and $\Delta\Delta C_T$ quantification; WT n=6 (except *MT-ND3* and *NDUFS3*, n=3), $TAZ^{\Delta 45}$ n=6 (except *MT-ND3* and *NDUFS3*, n=3), CTRL n=10 (except *MT-ND3* n=5) Significant differences are indicated; * ≤ 0.05, ** ≤ 0.005

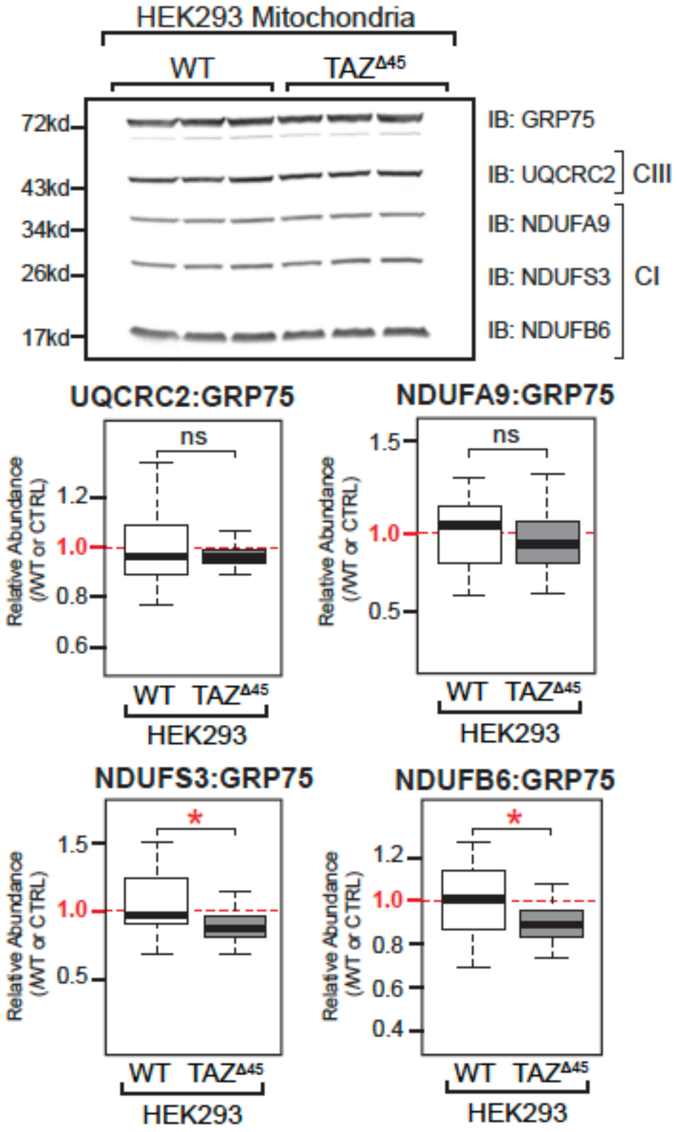

Figure S5. Immunoblotting of isolated mitochondria for CI and CIII subunits. Mitochondria (40 µg) isolated from the indicated lines were immunoblotted for the indicated proteins. Band intensities, relative to loading control GRP75, were quantified and plotted relative to WT/CTRL abundance; WT n=15, *TAZ*$^{\Delta45}$ n=15. Significant differences are indicated; * ≤ 0.05, ** ≤ 0.005.

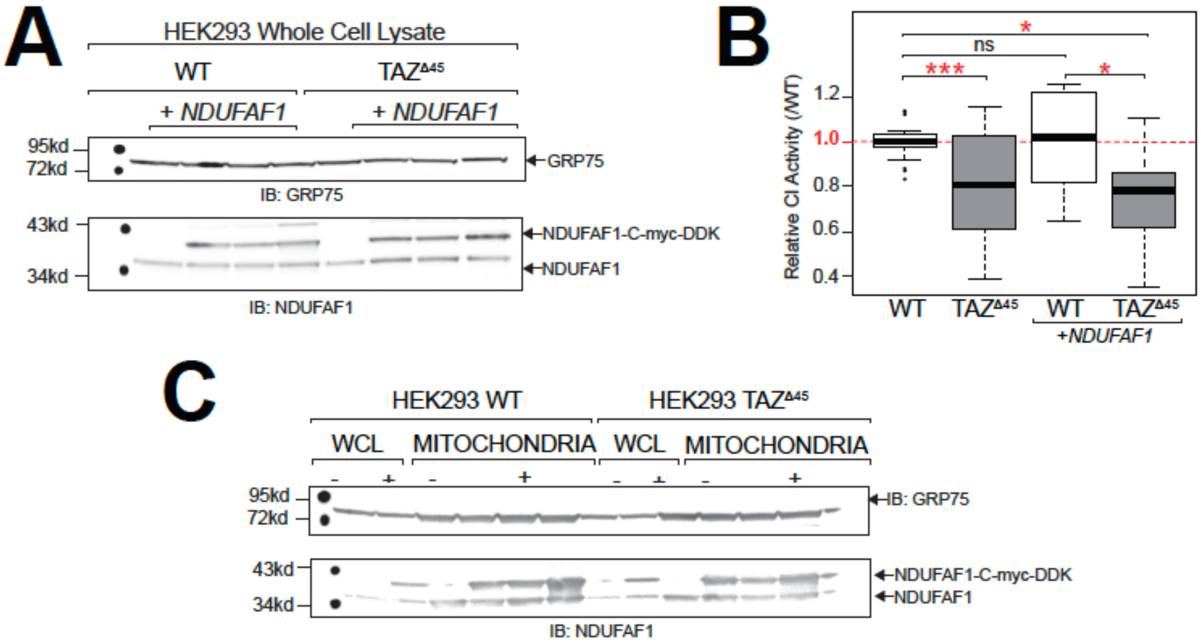

Figure S6. Overexpression of CI assembly factor NDUFAF1 does not normalize CI activity. **(A)** HEK293 WT and *TAZ*$^{\Delta 45}$ cells were transiently transfected with tagged *NDUFAF1* with Lipofectamine 3000 according to manufacturer's instructions. Whole cell extracts (45 µg) of the indicated lines and treatment concentrations were immunoblotted for the indicated proteins. **(B)** CI activity measured in mitochondria (200 µg total protein). Activity was measured on a microplate reader (450nm) by following the oxidation of NADH to oxidized nicotinamide adenine dinucleotide (NAD+). Activity plotted relative to WT abundance; WT n=25, *TAZ*$^{\Delta 45}$ n=26, WT-transfected n=9, *TAZ*$^{\Delta 45}$ n=8. (**C**) HEK293 WT and *TAZ*$^{\Delta 45}$ cells were transiently transfected with tagged *NDUFAF1* with Lipofectamine 3000 according to manufacturer's instructions. Whole cell extracts and isolated mitochondria (45 µg) of the indicated lines and treatment concentrations were immunoblotted for the indicated proteins. Significant differences are indicated; * ≤ 0.05, ** ≤ 0.005, *** ≤ 0.0005, **** ≤ 0.00005.

Table S4. Abundance of PARL with CCCP treatment (20uM) at serial time points (Figure 3E).

Cell Line	Time Point	n#	Relative Abundance (/WT 0 mins)	WT vs. *TAZ*$^{\Delta 45}$ For each time point
WT	0 MIN	54	1.00	p= 1.8 x 10^{-10}
TAZ$^{\Delta 45}$		48	1.51	
WT	10 MIN	5	1.13	p= 1.3 x 10^{-3}
TAZ$^{\Delta 45}$		5	1.48	
WT	30 MIN	6	1.11	p= 2.5 x 10^{-3}
TAZ$^{\Delta 45}$		5	1.44	
WT	60 MIN	6	1.14	p= 6.4 x 10^{-3}
TAZ$^{\Delta 45}$		5	1.44	
WT	90 MIN	5	0.99	p= 5.7 x 10^{-5}
TAZ$^{\Delta 45}$		5	1.38	
WT	120 MIN	6	1.14	ns
TAZ$^{\Delta 45}$		6	1.42	

Table S5. Percent of PGAM5 cleavage with CCCP treatment (20uM) at serial time points (Figure 3D).

Cell Line	Time Point	n#	Percent (%) Cleaved PGAM5	WT vs. *TAZ*$^{\Delta45}$ For each time point	Difference (%) in Percent Cleaved
WT	0 MIN	41	12	p= 1.5 x 10^{-7}	11
TAZ$^{\Delta45}$		41	23		
WT	10 MIN	8	11	ns	9
TAZ$^{\Delta45}$		8	20		
WT	30 MIN	8	18	p= 0.001	15
TAZ$^{\Delta45}$		7	33		
WT	60 MIN	8	29	p= 3.0 x 10^{-5}	16
TAZ$^{\Delta45}$		5	45		
WT	90 MIN	5	39	p= 2.4 x 10^{-4}	18
TAZ$^{\Delta45}$		7	57		
WT	120 MIN	7	49	p= 7.5 x 10^{-5}	18
TAZ$^{\Delta45}$		7	67		

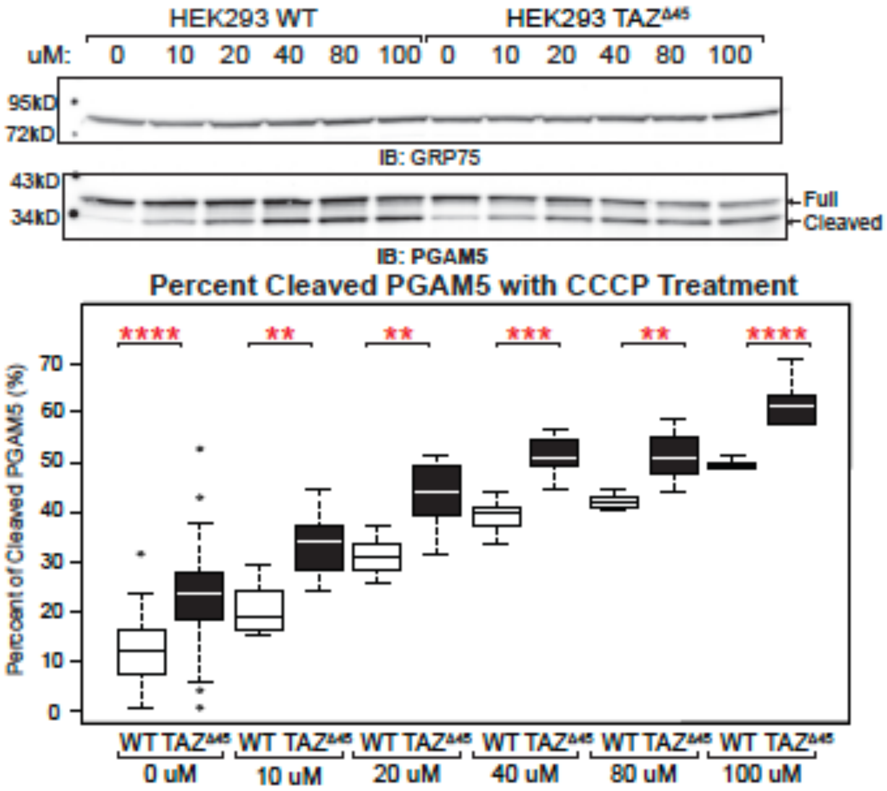

Figure S7. Increased PGAM5 cleavage with increasing levels of CCCP concentration in *TAZ*$^{\Delta 45}$ cells. HEK293 WT and *TAZ*$^{\Delta 45}$ cells were treated for 45 minutes with the indicated concentrations. Whole cell extracts (45 µg) of the indicated lines and treatment concentrations were immunoblotted for the indicated proteins. Band intensities, relative to the loading control GRP75, for both full-length and cleaved PGAM5 were individually quantified and plotted as the percent of cleaved PGAM5 (cleaved/full+cleaved); WT n=18, *TAZ*$^{\Delta 45}$ n=18, per treatment. Significant differences are indicated; * ≤ 0.05, ** ≤ 0.005, *** ≤ 0.0005, **** ≤ 0.00005.

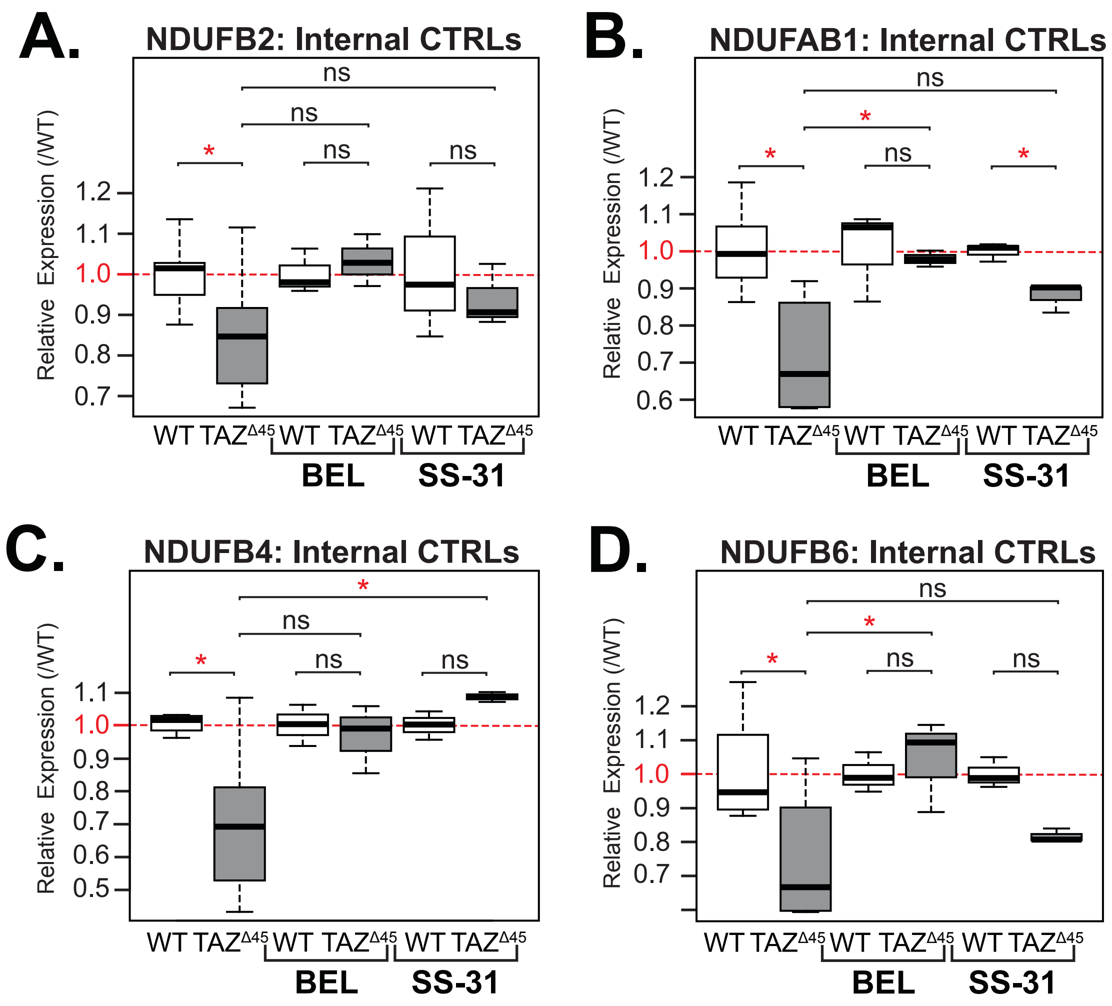

Figure S8. Relative mRNA expression of (A) *NDUFB2* (B) *NDUFAB1* (C) *NDUFB4* and (D) *NDUFB6* after treatment with BEL and SS-31. Expression determined by qRT-PCR and $\Delta\Delta C_T$ quantification using each respective control; WT n=6, *TAZ*$^{\Delta45}$ n=3, WT-BEL n=3, *TAZ*$^{\Delta45}$-BEL n=3, WT-SS-31 n=3, *TAZ*$^{\Delta45}$-SS-31 n=3 per gene. Significant differences are indicated; * ≤ 0.05, ** ≤ 0.005, *** ≤ 0.0005, **** ≤ 0.00005.

Table S6. Primers used for qRT-PCR.

Gene	Sequence (5' – 3')	
TBP	Forward	GAGCTGTGATGTGAAGTTTCC
	Reverse	TCTGGGTTTGATCATTCTGTAG
HPRT1	Forward	TGAGGATTTGGAAAGGGTGT
	Reverse	GAGCACACAGAGGGCTACAA
NDUFA9	Forward	CGCATGGGGTCACAGGTAAT
	Reverse	CTCGCGTCCCATTCCAGAAA
NDUFS3	Forward	TACACAGATGAGCTGACGCC
	Reverse	TCCAAACATGTCCCAGATCTCC
MT-ND3	Forward	ACTACCACAACTCAACGGCT
	Reverse	GCGGGGGATATAGGGTCGAA
NDUFB4	Forward	CATGGGAGCTCTGTGTGGAT
	Reverse	TTCTTTCCTATCCCTCTCAGTTTT
NDUFAB1	Forward	GCCGCCAGTATAGCGACAT
	Reverse	CCAAACTGTCTAAGCCCAGGT
NDUFA5	Forward	GCGGGTGTGCTGAAGAAGA
	Reverse	TTCCGCTTTAACCATAGCCAG
NDUFB2	Forward	GAACTCGCTCTGGAACACCT
	Reverse	ACTGCTGAAGATGGTGGAGT
NDUFB6	Forward	TCCATGGGGTATACAAAAAGAG
	Reverse	GGAAATTCTTTCATTGGTGGA
NDUFAF1	Forward	GGCAGGAGGTCAAGATTCCTT
	Reverse	AGCCAAGGTGAATCCTATAGAAGAG
PARL	Forward	CGCCATGGATACAGCAGGA
	Reverse	CACTAGCGGCTCCCTGTTCTT
MT-RNR1	Forward	TAGAGGAGCCTGTTCTGTAATCGAT
	Reverse	CGACCCTTAAGTTTCATAAGGGCTA
MT-CO1	Forward	GACGTAGACACACGAGCATATTTCA
	Reverse	AGGACATAGTGGAAGTGAGCTACAAC
MT-ATP6	Forward	TAGCCATACACAACACTAAAGGACGA
	Reverse	GGGCATTTTTAATCTTAGAGCGAAA
